# Systematic comparison of inverse Langevin function approximations in stochastic dumbbell dynamics

**DOI:** 10.1016/j.jnnfm.2026.105610

**Published:** 2026-04-28

**Authors:** Michael Cromer, Paula A. Vasquez

**Affiliations:** a School of Mathematics and Statistics, Rochester Institute of Technology, Rochester, NY 14623, USA; b Department of Mathematics, University of South Carolina, Columbia, SC 29208, USA

**Keywords:** Brownian configuration fields, Inverse Langevin function, Elastic dumbbells

## Abstract

Finite extensibility strongly influences the nonlinear dynamics of dilute polymer solutions, yet its representation in stochastic dumbbell models typically relies on approximate forms of the inverse Langevin function. Here, we systematically examine how commonly used inverse Langevin function approximations affect both the model predictions and numerical behavior of stochastic dumbbell models. Using the Brownian Configuration Field formulation, we directly integrate the full stochastic equations for finitely extensible dumbbells and compare three spring-force representations: the classical Finitely Extensible Nonlinear Elastic (FENE) model and two Padé-based approximations due to Cohen and Rickaby–Scott. Model predictions are assessed in large-amplitude oscillatory shear, steady uniaxial extensional, and capillary thinning flows for different parameter values. The results show that differences emerge when chains remain at moderate extension, whereas weakly deformed chains and chains rapidly driven to near-full extension exhibit model-independent behavior. In these transitional regimes, the FENE model consistently predicts lower stretch and stress levels than the Padé-based approximations, with discrepancies increasing for highly extensible chains. Analysis of the governing equations further demonstrates that the choice of approximation controls the stiffness of the stochastic dynamics near full extension, directly impacting numerical stability in coupled flow simulations. These results indicate that the choice of inverse Langevin approximation can measurably affect both model predictions and numerical robustness in stochastic simulations of nonlinear viscoelastic flows.

## 1. Introduction

Dilute polymer solutions consist of long-chain molecules dispersed in a solvent at low concentrations, where interactions between different chains are negligible. Despite this apparent simplicity, such systems exhibit a rich variety of flow behaviors. Individual polymer molecules can stretch, rotate, and align under deformation, and these microscopic motions give rise to macroscopic effects such as drag reduction in pipelines and beads-on-a-string formation during droplet breakup. Understanding how molecular configurations influence bulk flow is therefore essential for predicting the viscoelastic behavior of polymeric fluids and for developing models that accurately represent their underlying physics.

To explore these couplings between molecular and macroscopic behavior, it is often useful to employ simplified representations that retain key physical mechanisms while remaining computationally tractable. In this work, we adopt the elastic dumbbell framework, in which each polymer chain is modeled as two beads connected by a spring. This minimal model captures the dominant relaxation mechanisms associated with chain stretching and orientation. Dumbbell models have long been used to study dilute polymer solutions because they strike a balance between analytical tractability and the ability to capture essential features of polymer dynamics under flow [1]. As emphasized in Kröger’s comprehensive review of micro- and mesoscopic models for polymeric fluids [2], such bead-spring descriptions form the foundation of polymer kinetic theory and reliably capture essential nonequilibrium behavior despite their simplicity. To simulate the collective behavior of many chains, we use the Brownian Configuration Field (BCF) method [3], a stochastic field-based approach that evolves ensembles of configuration fields governed by Langevin-type equations. Ensemble averages of these fields yield macroscopic quantities such as the conformation tensor and polymeric stress, enabling flow simulations that incorporate essential microstructural dynamics without requiring the explicit tracking of individual molecules.

A key physical ingredient in these models is finite chain extensibility, which sets the maximum elongation a polymer chain can achieve under flow. The importance of finite extensibility in bead-spring models and extensional rheology has long been recognized and is discussed extensively in standard references on polymer kinetic theory and rheology [1,4,5]. This feature becomes particularly important in strong extensional or high-shear regimes, where chains approach their full contour length. Classical molecular simulations have shown that during rapid stretching, the effective spring force can exhibit pronounced history dependence—an effect not captured by the standard FENE dumbbell model [6]. Because the finite extensibility limit strongly influences polymer alignment, stress growth, and flow stability, it is a central ingredient in describing nonlinear viscoelastic behavior.

Experimental studies have provided direct evidence of these effects across a range of flow configurations. In stagnation-point flows, Haward et al. [7,8] showed that once the finite extensibility limit is reached, the flow field stabilizes and the birefringence strand saturates, providing direct optical confirmation of the maximum chain extension. In capillary thinning experiments, Dinic and Sharma [9] demonstrated that for high-molecular-weight polymers the filament thinning rate changes markedly when chains reach their extensibility limit, giving rise to a terminal viscoelastic capillary (TVEC) regime governed by finite extensibility. Clasen et al. [10] further showed that finite extensibility cannot be neglected even in dilute solutions during the short intermediate elastocapillary thinning regime, with apparent relaxation times and self-concentration effects that depend sensitively on chain length. Oliveira et al. [11] observed experimentally that finite extensibility truncates the iterated beads-on-a-string instability in high-molecular-weight polymer solutions, halting the recursive formation of successive bead generations and driving progressive coalescence prior to filament rupture. Calabrese et al. [12] further reported that highly extensible polymers exhibit enhanced alignment and stress anisotropy in capillary-driven extensional flows, whereas less extensible chains show delayed transitions to elastocapillary thinning and apparent underestimation of relaxation times. Together, these findings underscore that the degree of extensibility is a decisive factor in shaping flow dynamics and stability.

More recent theoretical and computational studies have likewise highlighted how finite extensibility governs flow stability and stress evolution. Khalid et al. [13] analyzed elastic instabilities in channel flows using the FENE-P and FENE-CR models and found that finite extensibility can either stabilize or destabilize the flow depending on the elasticity number, with the FENE-P model tending to promote instability at high elasticity due to enhanced stress anisotropy, while the FENE-CR model exhibits a more uniform relaxation response that mitigates such effects. Zhang et al. [14] compared modal and non-modal instabilities in channel flows modeled by Oldroyd-B and FENE-P fluids, showing that finite extensibility alters the coupling between velocity perturbations and polymer stress. Beneitez et al. [15] identified a polymer diffusive instability in inertialess shear flows, where small stress diffusivities can give rise to chaotic dynamics reminiscent of elastic turbulence. Yamani and McKinley [16] examined steady shear flows of FENE-P fluids and demonstrated that the ratio of the Weissenberg number to the finite extensibility parameter (Wi/b) determines when finite extensibility effects become dominant, with strong deviations occurring once Wi/b≥1. Zinelis et al. [17,18] showed numerically that finite extensibility interacts with initial stress distributions generated by pre-shear in nozzles, promoting beads-on-a-string formation and delaying filament breakup in jetting flows. These studies collectively reveal that finite extensibility is a fundamental parameter controlling the stability, stress evolution, and nonlinear dynamics of viscoelastic flows, especially when polymer chains approach their maximum extension.

Although the physical importance of finite extensibility is well established, the way in which its mathematical representation affects stochastic dumbbell dynamics remains less well understood. It is useful here to distinguish two related questions in the literature. One concerns the coarse-graining of detailed polymer models into multibead bead-spring chains and the extent to which different nonlinear spring laws reproduce force-extension behavior and strong-flow predictions [19–22]. The other, which is the focus of the present work, concerns how the representation of finite extensibility influences predictions within a fixed stochastic dumbbell model. We address this latter question within a free-draining dumbbell/BCF framework. Most theoretical and computational models rely on constitutive closures that approximate ensemble-averaged behavior, such as the FENE-P and FENE-CR models. However, these closures introduce assumptions that can obscure the direct influence of finite extensibility on molecular configurations. To isolate these effects, we solve the full stochastic dumbbell equations within the BCF framework, without invoking constitutive closures. Here, BCF is used as a methodological choice rather than a unique one: it is one of several closure-free micro–macro approaches, alongside direct Brownian dynamics simulations, Fokker–Planck solvers, and related formulations. We adopt it because it provides a numerically efficient field-based framework that yields smooth stress fields and is well suited to systematic comparisons of spring-force representations in coupled flow simulations. In this setting, our goal is to compare inverse Langevin approximations within a common dumbbell model, not to recalibrate the models to enforce identical equilibrium or linear viscoelastic properties, nor to remove the broader limitations of the underlying free-draining dumbbell description [1,2,23]. This approach allows us to assess how the representation of finite extensibility affects both model predictions and numerical behavior within the stochastic dumbbell model. Earlier studies have shown that closure approximations can lead to significant deviations from the underlying stochastic dumbbell dynamics. Herrchen and Öttinger [24] demonstrated that different closure schemes produce distinct stress and relaxation responses even under identical flow conditions, and Vincenzi et al. [25] showed that the Peterlin approximation in FENE-P tends to overestimate chain extension and underpredict orientation correlations in turbulent flows. By bypassing such approximations, our approach directly examines how finite extensibility, as represented in the dumbbell force law, influences predictions within the underlying stochastic dumbbell model.

The finite extensibility of a dumbbell spring is typically described through the inverse Langevin function (ILF), which relates the entropic restoring force to the fractional chain extension [5,26–28]. This formulation originates from the statistical mechanics of freely-jointed chains and provides a physically grounded description of nonlinear elasticity. However, the ILF lacks a closed-form expression and becomes increasingly stiff as the chain approaches full extension, posing challenges for both analysis and computation. To overcome these difficulties, a variety of analytical approximations have been proposed, including truncated series expansions and rational (Padé) forms, each offering different trade-offs between accuracy, computational cost, and numerical stability [27,29–34].

In this work, we systematically compare how *three* different ILF approximations affect the behavior of stochastic dumbbell models within the BCF framework. Throughout this study, the FENE, Cohen, and RS models are compared at fixed spring parameters, so the observed differences are interpreted as consequences of the inverse Langevin approximation itself within a common dumbbell framework rather than of a separate recalibration step. By comparing their performance across diverse flow conditions, we assess how these approximations influence polymer stretching, stress evolution, and numerical stability. Our analysis shows that the choice of ILF approximation can significantly alter both the model predictions and the convergence properties of simulations. These findings show that the inverse Langevin representation is not merely an implementation detail, but a consequential modeling choice within this framework.

The remainder of this paper is organized as follows. Section 2 presents the governing equations for finitely extensible stochastic dumbbell models and introduces the three spring-force representations considered here (FENE, Cohen, and Rickaby–Scott). Section 3 compares the predictions of the three models in viscometric flows, including large-amplitude oscillatory shear and uniaxial extension. Section 4 examines a non-viscometric flow problem, namely capillary thinning of a viscoelastic filament. Finally, Section 5 summarizes the main conclusions.

## 2. Governing equations

### 2.1. Macroscopic flow equations

At the macroscale, considering an unsteady, incompressible viscoelastic flow in the absence of body forces, the flow is governed by the conservation of mass and momentum equations:

(1) $$\tilde{\nabla }\cdot \tilde{u}=0,$$

(2) $$\rho \left(\frac{\partial \tilde{u}}{\partial \tilde{t}}+\tilde{u}\cdot \tilde{\nabla }\tilde{u}\right)=-\tilde{\nabla }\tilde{p}+\eta_{s}{\tilde{\nabla }}^{2}\tilde{u}+\tilde{\nabla }\cdot \tilde{\tau },$$

where $\tilde{u}$ is the velocity vector, $\tilde{p}$ the pressure, $\tilde{\tau }$ the extra stress tensor, $\eta_{s}$ the solvent viscosity, and ρ the density.

We nondimensionalize these equations using the following scaling:

(3) $$t=\tilde{t}\cdot \left(\frac{1}{\;\text{T}}\right),\;x=\tilde{x}\cdot \left(\frac{1}{\;\text{L}}\right),\;u=\tilde{u}\cdot \left(\frac{1}{\text{U}}\right),\;p=\tilde{p}\cdot \left(\frac{1}{\rho {\text{U}}^{2}}\right),\;\tau =\tilde{\tau }\cdot \left(\frac{\lambda }{\eta_{0}}\right).$$

Here, T,L, and U are, respectively, the *macroscopic* characteristic time, length, and velocity scales, related by U=L/T. In addition, $\eta_{0}$ denotes the zero-shear-rate viscosity of the fluid, and λ denotes the longest relaxation time of the polymeric component. Applying this scaling yields the dimensionless conservation equations:

(4a) ∇⋅u=0,

(4b) $$\frac{\partial u}{\partial t}+u\cdot \nabla u=-\nabla p+\frac{1}{Re}\left[\beta \nabla^{2}u+\frac{1}{De}\nabla \cdot \tau \right].$$

This formulation introduces several important dimensionless groups:

- The Reynolds number, $Re=\frac{\rho \text{UL}}{\eta_{0}}$, representing the ratio of inertial to viscous forces.
- The viscosity ratio, $\beta =\eta_{s}/\eta_{0}$, indicating the proportion of solvent viscosity to total zero-shear viscosity, $\eta_{0}=\eta_{s}+\eta_{p}$.
- The Deborah number is defined here as De=λU/L where the characteristic flow time is taken as L/U; it measures the ratio of the fluid relaxation time to the flow-time scale. In steady viscometric shear flows, where U/L may be identified with the shear rate ${\dot{\gamma }}_{0}$, this definition reduces to the familiar Weissenberg number $\text{Wi}=\lambda {\dot{\gamma }}_{0}$.

To close the system of governing equations (Eq. (4)), a constitutive relation linking the extra stress tensor, τ, to the microscopic polymer configuration is required. In polymeric fluids, this stress arises from the configuration and dynamics of polymer chains suspended in a solvent. Many constitutive models have been developed to describe this coupling [1,4,35], ranging from continuum-based differential formulations to kinetic-theory approaches. Here, we adopt a mesoscopic framework derived from polymer kinetic theory, in which the microstructure is represented by ensembles of coarse-grained elastic dumbbells.

### 2.2. Dumbbell models

The dumbbell model idealizes a flexible polymer chain as two beads connected by an entropic spring. In this approach, the solvent is assumed to be an incompressible Newtonian fluid with viscosity $\eta_{s}$. The configuration of a dumbbell is described by the end-to-end connector vector $Q=r_{2}-r_{1}$ and the center-of-mass vector $r_{c}=\frac{1}{2}\left(r_{1}+r_{2}\right)$. The stochastic differential equation describing the evolution of the end-to-end vector is given by [1,23],

(5) $$d{\tilde{Q}}_{t}=(\tilde{\nabla }\tilde{u}{)}^{⊤}\cdot {\tilde{Q}}_{t}-\frac{2}{\zeta }F({\tilde{Q}}_{t})+\sqrt{\frac{4k_{B}T}{\zeta }}dW_{t},$$

where F(⋅) denotes the spring force connecting the two beads, $W_{t}$ is a Wiener process of the same dimension as Q,ζ is the drag coefficient acting on each bead, and $k_{B}T$ is the thermal energy.

The viscoelastic contribution to the stress is given by the Kramers relation [1,23],

(6) $$\tilde{\tau }=n\left\langle F\left(\tilde{Q}\right)\tilde{Q}\right\rangle -nk_{B}TI,$$

where n is the dumbbell number density, n⟨F(Q)Q⟩ represents the contribution from the spring tension with spring law F(Q), and $nk_{B}T$ accounts for Brownian motion effects.

To scale these equations, in addition to the relations in Eq.(3), we define a characteristic *microscopic* length scale in terms of the spring constant H:

$${\text{L}}_{\text{micro}}=\sqrt{\frac{k_{B}T}{H}},\;\text{so}\;\text{that}\;Q=\tilde{Q}\cdot \sqrt{\frac{H}{k_{B}T}},$$

and the longest relaxation time is defined as

(7) $$\lambda =\frac{\zeta }{4H}.$$

The resulting nondimensional equations for the dumbbell evolution and polymeric stress are

(8a) $$dQ_{t}=(\nabla u{)}^{⊤}\cdot Q_{t}-\frac{1}{2De}F\left(Q_{t}\right)+\frac{1}{\sqrt{De}}dW_{t},$$

(8b) $$\tau =\left(1-\beta \right)\left[\left\langle F\left(Q\right)Q\right\rangle -I\right],$$

where we have used the fact that $\eta_{p}=nk_{B}T\lambda$. To complete the formulation, it remains to specify the functional form of the spring force F(Q), which represents the elastic response of the polymer chain.

#### 2.2.1. Spring force

To derive a physically motivated spring force, we appeal to the statistical mechanics of freely-jointed chains. Consider a polymer chain composed of $N_{K}$ Kuhn segments of length $\ell_{K}$, giving a total contour length $L_{c}=N_{K}\ell_{K}$. For such a chain subjected to an applied force F, the freely-jointed chain (FJC) model relates the mean fractional extension $x=\langle r\rangle /L_{c}$ to the dimensionless force $\xi =F\ell_{K}/\left(k_{B}T\right)$ through the Langevin function [5,28]:

(9) $$x=ℒ\left(\xi \right)=\text{coth}\left(\xi \right)-\frac{1}{\xi }.$$

In the dumbbell model, the end-to-end vector Q plays the role of the instantaneous chain extension, with fractional extension $x=Q/Q_{\text{max}}$ where $Q_{\text{max}}=L_{c}$. The entropic spring force is then obtained by inverting Eq. (9), expressing force as a function of the current extension:

(10) $$\xi ={ℒ}^{-1}\left(x\right).$$

The inverse Langevin function, ${ℒ}^{-1}$, is therefore the core component in specifying the entropic spring force F(Q) for the dumbbell model. It captures the essential feature of entropic stiffening as polymer chains approach their contour length [1,5,26,28,36]. In the dumbbell representation, the fractional extension $x=Q/Q_{\text{max}}$ corresponds to the polymer chain’s fractional extension $\langle r\rangle /\left(N_{K}\ell_{K}\right)$, with $Q_{\text{max}}=N_{K}\ell_{K}=L_{c}$. Accordingly, the connector force F(Q) is given by the relation:

(11) $$F\left(Q\right)=\frac{k_{B}T}{\ell_{K}}{ℒ}^{-1}\left(\frac{Q}{Q_{\text{max}}}\right)\overset{ˆ}{Q}.$$

Here, Q=‖Q‖ and $\overset{ˆ}{Q}=Q/Q$ is the unit vector in the direction of Q. Defining the entropic spring constant as,

(12) $$H=\frac{3k_{B}T}{N_{K}\ell_{K}^{2}}=\frac{3k_{B}T}{Q_{\text{max}}\ell_{K}},$$

we obtain,

(13) $$F\left(Q\right)=\frac{HQ_{\text{max}}}{3}{ℒ}^{-1}\left(\frac{Q}{Q_{\text{max}}}\right)\overset{ˆ}{Q}.$$

Eq. (13) provides a general formulation of the entropic spring force, where the nonlinear elasticity is governed by the inverse Langevin function (ILF).

The ILF is defined for x∈(0,1). As x→0, the function behaves linearly, ${ℒ}^{-1}(x)\approx 3x+𝒪\left(x^{3}\right)$, corresponding to the Hookean regime. At the other extreme, as $x\to 1^{-}$, the function diverges, ${ℒ}^{-1}(x)\to \infty$, reflecting the finite extensibility of the polymer chain. Because ${ℒ}^{-1}(x)$ is transcendental and cannot be expressed in terms of elementary functions, it lacks a closed-form analytical representation and is costly to evaluate numerically. To overcome these limitations, a wide range of approximations have been developed [27,29–34,37–48]. Among them, two classes are most commonly used in polymer modeling: series expansions and Padé approximants.

Series-type approximations for the ILF were first introduced by Kuhn and Grün [26] as one of the earliest approaches to describe chain elasticity. These early power-series expansions, later formalized as Taylor-series truncations about small extensions (x≈0) [37], provide accurate results over moderate deformation ranges. However, they exhibit poor convergence near the singular point x=1, corresponding to highly stretched chains. In fact, convergence typically breaks down near x≈0.904 because of complex-plane singularities [44].

In contrast, Padé approximations provide a more versatile and accurate alternative. Their rational functional form allows simple implementation while correctly reproducing the asymptotic behavior of the ILF as $x\to 1^{-}$ [30]. Owing to this balance between simplicity and accuracy, Padé-type expressions remain the most widely used in analytical and computational polymer models [2,27,29,39,41,49].

In what follows, we use the general force expression in Eq.(13) to construct *three* specific dumbbell models, each corresponding to a different approximation of the inverse Langevin function.

**Fig. 1.**
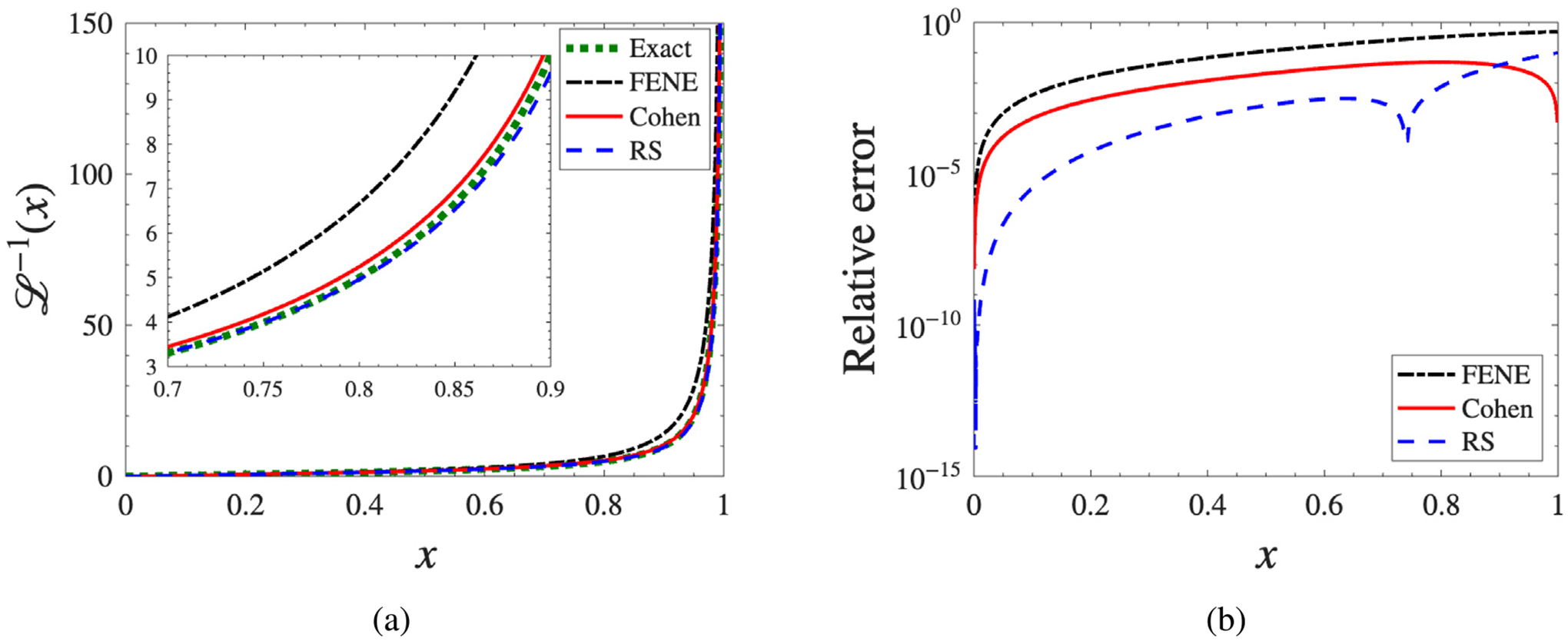
Approximations vs exact: (a) direct comparison of approximants with the exact inverse Langevin function; (b) relative error of each approximant as a function of extension.

1. *FENE spring law* The most widely used approximation is that proposed by Warner [27], which yields the Finitely Extensible Non-linear Elastic (FENE) spring law. As emphasized in Kröger’s review of micro- and mesoscopic polymer models [2], the FENE form has become the canonical representation of finite extensibility in bead-spring dumbbells due to its simplicity, its ability to reproduce the essential nonlinear stiffening of stretched chains, and its historical role as the simplest nonlinear elastic surrogate of freely-jointed chain statistics. The approximation
(14) $${ℒ}_{\text{FENE}}^{-1}(x)\approx \frac{3x}{1-x^{2}},$$
  can be retrospectively viewed as a closed-form surrogate for the inverse Langevin function. Although Warner originally introduced this spring force phenomenologically, the FENE form represents a nonlinear extension of the linear approximation ${ℒ}^{-1}(x)\approx 3x$, with the denominator $\left(1-x^{2}\right)$ accounting for finite chain extensibility. This modification retains the correct Hookean limit as x→0 while capturing the strong stiffening as $x\to 1^{-}$ [1,27]. With this approximation, Eq. (13) becomes
(15) $$F\left(\tilde{Q}\right)=\frac{H\tilde{Q}}{1-{\left(\tilde{Q}/Q_{\text{max}}\right)}^{2}}.$$
2. *Cohen [3/2] Padé approximation*
  Cohen [29] developed a Padé approximant that closely matches the inverse Langevin function over the full range of extensions. A [M/N] Padé approximant represents a function as a ratio of two polynomials, $P_{M}(x)/Q_{N}(x)$, where $P_{M}(x)$ and $Q_{N}(x)$ have degrees M and N, respectively, and $Q_{N}(0)=1$ for normalization. The coefficients are chosen so that the error $f(x)-\frac{P_{M}(x)}{Q_{N}(x)}$ is of order $O\left(x^{M+N+1}\right)$, ensuring high accuracy near x=0. Using this approach, Cohen proposed the following [3/2] Padé approximant for the inverse Langevin function:
(16) $${ℒ}_{\text{Coh}}^{-1}\left(x\right)\approx 3x\frac{1-\frac{1}{3}x^{2}}{1-x^{2}}.$$
  This rational form captures the singularity structure of the exact function at x=1 and maintains finite extensibility. Kröger [2] reports that Cohen’s approximation achieves a maximum relative error of 4.94%, compared to 50% for the FENE model.
  For the Cohen approximation, Eq.(13) becomes
(17) $$F(\tilde{Q})=\frac{1-{\left(\tilde{Q}/\left(\sqrt{3}\;Q_{\text{max}}\right)\right)}^{2}}{1-{\left(\tilde{Q}/Q_{\text{max}}\right)}^{2}}(H\tilde{Q}).$$
3. *Rickaby and Scott Padé approximation of the reduced ILF*
  Rickaby and Scott proposed a model based on Padé approximants of the Reduced Inverse Langevin Function (RILF) [39]. The RILF is constructed by multiplicatively removing the two simple poles of the ILF at x=±1,
(18) $$\text{RILF}\left(x\right)=\left(1-x^{2}\right)\frac{{ℒ}^{-1}\left(x\right)}{3x}.$$
  This process yields a function that is finite at x=±1, which significantly improves approximation accuracy, particularly at high extensions where $x\to 1^{-}$.
  The authors obtain a [2/0] Padé approximation of the RILF, $\text{RILF}(x)\approx 1-\frac{2}{5}x^{2}$, which is then multiplied by $\frac{3x}{1-x^{2}}$ to recover the ILF approximant:
(19) $${ℒ}_{\text{RS}}^{-1}\left(x\right)\approx 3x\frac{1-\frac{2}{5}x^{2}}{1-x^{2}}.$$
  Rickaby and Scott demonstrated that their model, hereafter denoted RS, significantly improves accuracy over Cohen’s approximation while maintaining comparable simplicity [39]. Across multiple test metrics — including stress response, strain energy, and various deformation modes — the RS model exhibits substantially lower mean percentage errors. For instance, the stress response error decreases from 3.01% (Cohen) to 1.90% (RS), and the strain energy error from 2.32% to 0.58%.
  With the approximation given in Eq. (19), the spring force from Eq. (13) becomes
(20) $$F(Q)=\frac{1-{\left(\sqrt{2/5}\;Q/Q_{\text{max}}\right)}^{2}}{1-{\left(Q/Q_{\text{max}}\right)}^{2}}(HQ).$$
4. *General form of the dumbbell spring force*
  A unified expression for the FENE, Cohen, and RS approximations has been proposed in [39,50] as
(21) $${ℒ}^{-1}\left(x\right)\approx 3x\left(1+\frac{2A}{3}\frac{x^{2}}{1-x^{2}}\right),$$
  where A=3/2 for FENE, A=1 for Cohen, and A=9/10 for RS. Using this form, the spring force in Eq.(13) becomes
(22) $$F\left(\tilde{Q}\right)=H\tilde{Q}\left(1+\frac{2A}{3}\frac{{\left(\tilde{Q}/Q_{\text{max}}\right)}^{2}}{1-{\left(\tilde{Q}/Q_{\text{max}}\right)}^{2}}\right).$$
  Nondimensionalizing as before gives
(23) $$F(Q)=Q\left(1+\frac{2A}{3}\frac{Q^{2}/b}{1-Q^{2}/b}\right),$$
  where the extensibility parameter is defined as $b=HQ_{\text{max}}^{2}/\left(k_{B}T\right)$. As shown in Fig. 1(a), all three approximations reproduce the qualitative behavior of the ILF at small extensions. At large extensions, however, the FENE form slightly overestimates the force compared with the other two approximations. The comparison illustrates how the FENE model extends the linear Hookean approximation by incorporating finite chain extensibility, while the Padé-based Cohen and RS models further refine this correction, achieving closer agreement with the exact ILF across the full extension range.

The corresponding relative errors $\left(\left|{ℒ}_{\text{Approx.}}^{-1}(x)-{ℒ}^{-1}(x)\right|/{ℒ}^{-1}(x)\right)$ are shown in Fig. 1(b). Among the three models, the FENE approximation exhibits the largest error over most of the extension range, followed by the Cohen model, while the RS approximation provides the lowest overall error. The RS curve exhibits a sharp dip in relative error around x≈0.7−0.8. This occurs because the RS rational approximant crosses the exact inverse Langevin function at that point (the numerator of the relative error vanishes), producing a local zero in the plotted error. The dip is therefore a consequence of the approximant’s algebraic form and the match of low-order series coefficients, not of any non-smooth behavior in the underlying force law.

Although FENE slightly overestimates the ILF at large extensions, the most consequential model differences in our flows arise at *moderate* extension, where the spring-law curvature differs most and before saturation fully masks those differences. We did not observe a distinct flow-level signature associated with the RS relativeerror dip near x≈0.7−0.8; within statistical variability, LAOS and steady-extension trends vary smoothly with the fractional extension x, and any localized crossing appears to be subsumed by the broader moderate-extension curvature differences among the three ILF approximations.

Another feature worth noting is that these approximations differ not only in their relative error, but also in whether they preserve the symmetry and asymptotic behavior of the ILF. In [32], Kröger noted that these approximants differ in qualitative properties: the FENE form preserves the correct symmetry of the inverse Langevin function but not its asymptotic behavior, whereas Cohen preserves the correct asymptotic behavior but not the symmetry. The Rickaby–Scott approximation likewise does not preserve the symmetry nor the asymptotic behavior; Kröger reported a maximum relative error of 9.9% for this form.

The unified form in Eq. (23) is not merely a notational convenience; it plays a central role in the design of this study. Because all three approximations share the same rational structure, the semi-implicit time-integration scheme described in the supplemental material and in [51–53] reduces the implicit corrector step to the same scalar cubic equation for each model (Eq. (S6)), with differences entering only through the parameter A. This ensures that any differences observed in the stochastic dynamics or macroscopic stress arise from the approximation itself rather than from variations in numerical treatment. ILF approximations that do not admit this unified form would require different solver strategies, complicating the model comparisons. We note that other simple inverse Langevin approximants with improved accuracy also exist, such as the admissible rational form $L_{011}$ discussed by Kröger [32]. Additionally, in practice, one may simply numerically calculate the exact ILF utilizing, for example, Newton’s method or interpolation [43]. However, such approximants/approximations do not preserve the unified cubic structure of the semi-implicit update employed in this study and would therefore require different numerical treatment, thereby introducing additional differences unrelated to the spring-force approximation itself.

## 3. Model predictions for viscometric flows

In viscometric flows, assuming a fully developed state implies that velocity and stress fields remain constant along the flow direction, eliminating spatial gradients on that axis. This simplification decouples the dumbbell equations (Eqs. (8)) from the conservation equations, allowing them to be solved independently. Here, we examine two canonical viscometric flows: large-amplitude oscillatory shear (LAOS) and uniaxial extension.

LAOS imposes large-amplitude, time-dependent shear that produces complex nonlinear stress responses. Although oscillatory reversal limits sustained chain stretching, the imposed strains are still substantial, making LAOS an important case for testing whether differences among ILF approximations influence predictions under large yet transient deformations. Uniaxial extension complements LAOS by subjecting polymer chains to sustained elongational deformation, driving them into the high-stretch regime where distinctions among the three ILF approximations become most evident (see Fig. 1). This flow therefore isolates the distinct predictions that emerge from each approximation when finite extensibility dominates.

### 3.1. Large-amplitude oscillatory shear

In LAOS, the material undergoes time-periodic shear deformation with a strain amplitude sufficient to probe the nonlinear viscoelastic regime. The imposed strain and strain rate are, respectively,

(24) $$\gamma (t)=\gamma_{0}\;\text{sin}\;t\;\text{and}\;\dot{\gamma }(t)=\gamma_{0}\;\text{cos}\;t,$$

where the dimensionless time is $t=\tilde{t}\omega$. The corresponding nondimensional stochastic evolution equation for the dumbbell configuration vector Q is

(25) $$dQ_{t}=\frac{Wi}{De}(\nabla u{)}^{⊤}\cdot Q_{t}-\frac{1}{2De}F\left(Q_{t}\right)+\sqrt{\frac{1}{De}}dW_{t},$$

where the Deborah number De=λω characterizes flow unsteadiness and the Weissenberg number $Wi=\lambda {\dot{\gamma }}_{0}\left({\dot{\gamma }}_{0}=\gamma_{0}\omega \right)$ quantifies the strength of imposed deformation. Their ratio,

(26) $$\frac{Wi}{De}=\gamma_{0},$$

defines the strain amplitude, with $\gamma_{0}\approx 1$ marking the nominal boundary between linear and nonlinear viscoelastic response [54,55]. The independent variation of Wi and De thus allows LAOS to decouple the effects of deformation *amplitude* from those of the deformation *timescale*. Along the diagonal Wi=De, polymer chains begin to probe the nonlinear curvature of the spring force while remaining well below their finite extensibility limit. This regime is particularly useful for isolating whether discrepancies among ILF closures originate from finite extensibility or from differences in extension-dependent relaxation dynamics.

**Fig. 2.**
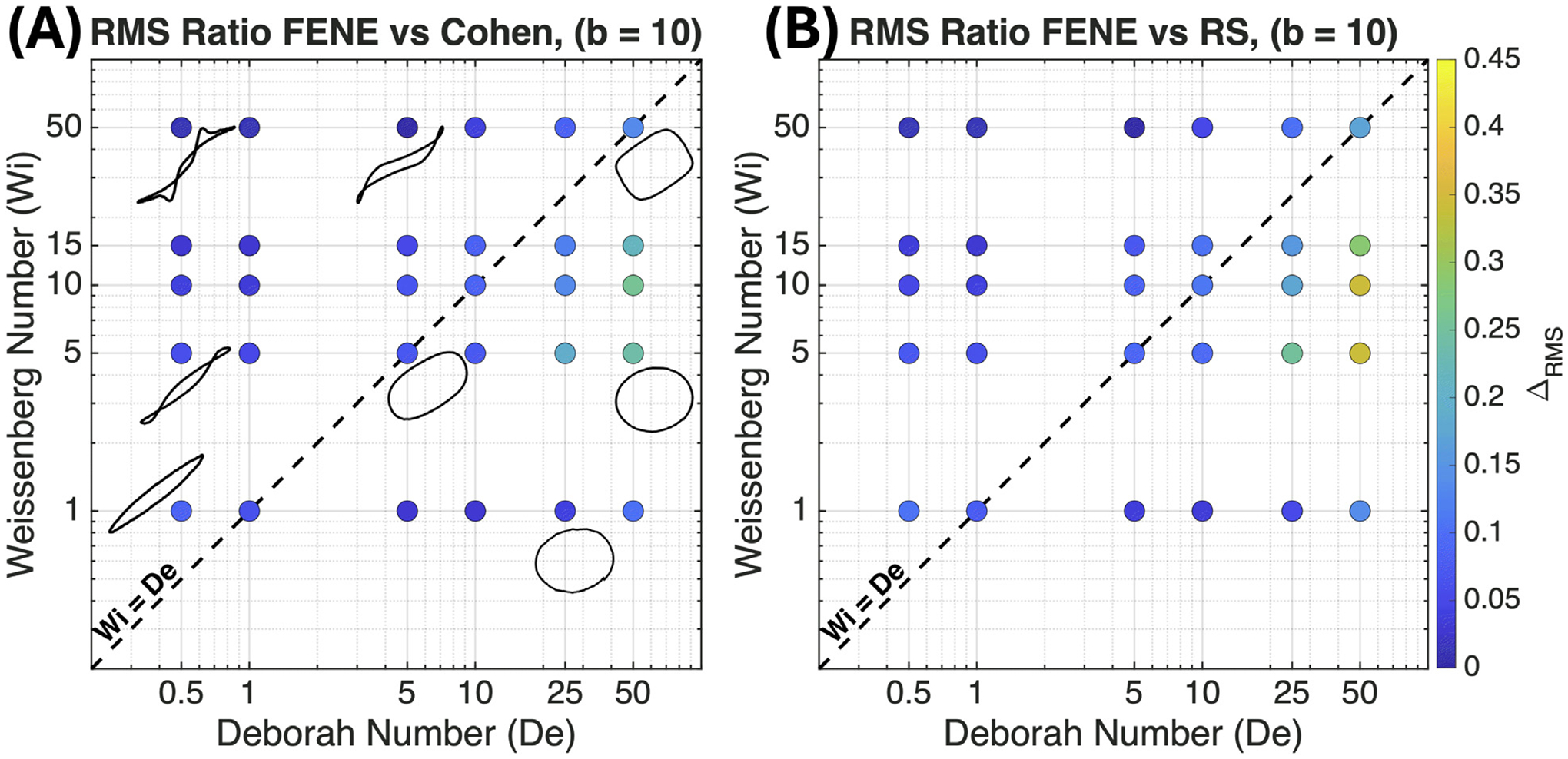
Pipkin diagrams illustrating the normalized cycle-averaged RMS difference, $\Delta_{\text{RMS}}$, in the third-harmonic ratio $\left(A_{3}/A_{1}\right)$ for extensible chains with b=10. Comparisons are shown between (A) FENE and Cohen models and (B) FENE and RS models. The color scale represents the relative deviation calculated via Eq. (27), with dark blue indicating close agreement. The dashed diagonal line (Wi=De) denotes a strain amplitude of $\gamma_{0}=1$. Representative viscous Lissajous–Bowditch curves are overlaid to contrast the elliptical response in the quasi-linear regime (bottom-right) with the highly distorted response in the large-amplitude regime (top-left).

**Fig. 3.**
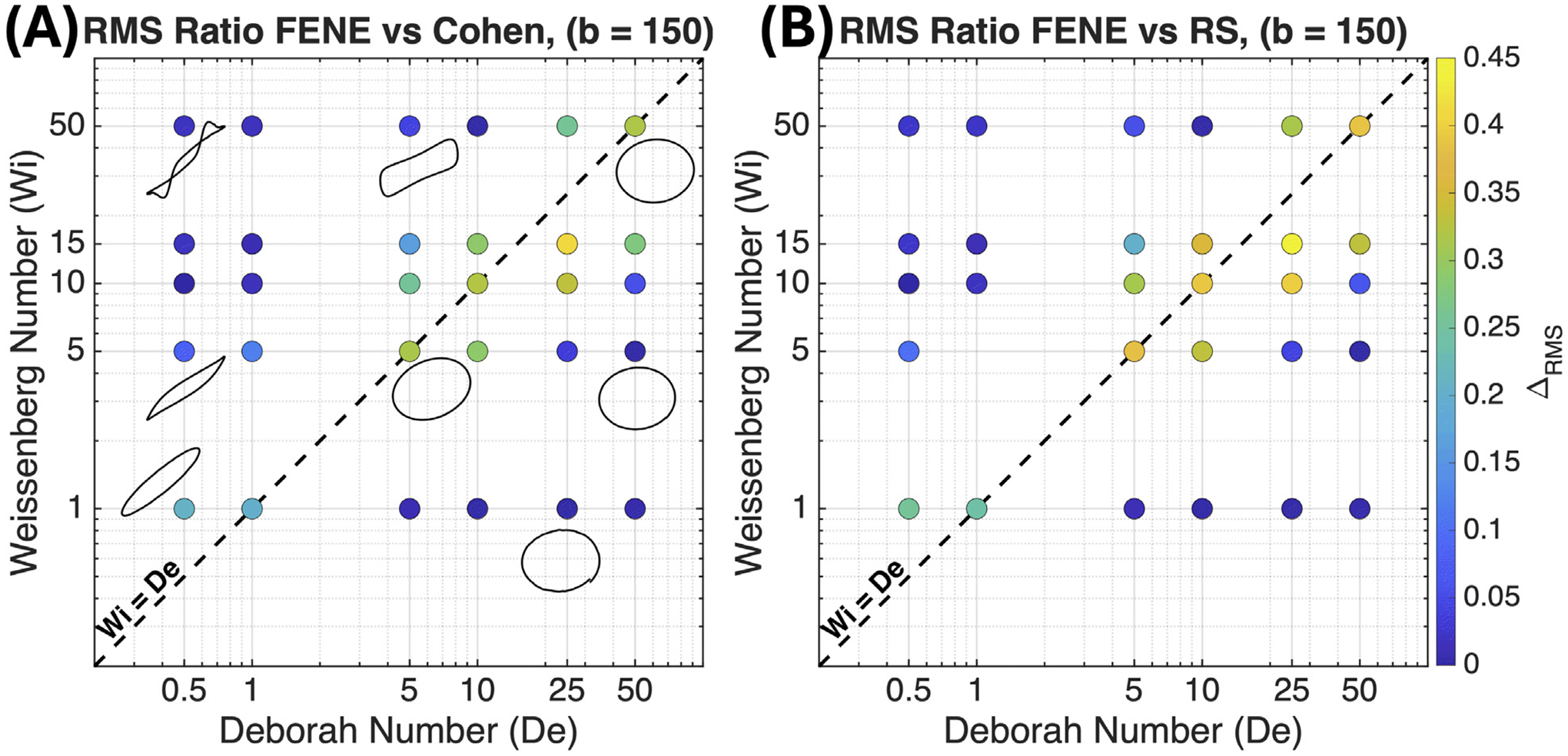
Pipkin diagrams illustrating the normalized cycle-averaged RMS difference, $\Delta_{\text{RMS}}$, in the third-harmonic ratio $\left(A_{3}/A_{1}\right)$. Comparisons are shown for finite extensibility parameter b=150 between (A) the FENE and Cohen models and (B) the FENE and RS models. The colorbar indicates the relative deviation, where $\Delta_{\text{RMS}}$ is calculated according to Eq. (27). The diagonal line (Wi=De) corresponds to a strain amplitude of $\gamma_{0}=1$.

To investigate these effects, we compute the steady-state oscillatory response over a wide range of (Wi,De) and summarize the results using Pipkin diagrams (Figs. 2 and 3). Model differences are measured using the third-harmonic ratio $A_{3}/A_{1}$, a standard LAOS metric associated with nonlinear intracycle chain stretching and nonlinear stress response [54,55]. To quantify deviations between the models, we employ the normalized root-mean-square (RMS) difference:

(27) $$\Delta_{\text{RMS}}\left(\frac{A_{3}}{A_{1}}\right)=\frac{1}{{\left\langle A_{3}/A_{1}\right\rangle }_{\text{FENE}}}\sqrt{\frac{1}{N}\sum_{i=1}^{N}{\left[{\left(\frac{A_{3}}{A_{1}}\right)}_{i,\text{model}}-{\left(\frac{A_{3}}{A_{1}}\right)}_{i,\text{FENE}}\right]}^{2},}$$

where the FENE model serves as the reference due to its role as the canonical finitely extensible dumbbell model [1]. The magnitude of $\Delta_{\text{RMS}}$ quantifies the degree to which a given ILF approximation (Cohen or RS) departs from the FENE reference.

We examine two contrasting extensibility limits, b=10 and b=150. The smaller value serves mainly as an illustrative low-extensibility/rapid-saturation limit, whereas the larger value is more representative of highly extensible chains and exposes a broader transition regime. In both cases, discrepancies among the ILF approximations are confined to transitional regimes of moderate chain stretch, where differences in spring-force curvature are most influential, and are suppressed in the two limiting regimes: the Hookean limit at weak deformation and the saturation limit at large strain amplitudes. The extensibility parameter b primarily controls the breadth of this transitional region. For small b, rapid approach to saturation restricts discrepancies to a narrow band of Pipkin space, whereas for large b, delayed saturation exposes a substantially broader range of flow conditions to curvature-dependent nonlinear effects.

#### 3.1.1. Small extensibility limit (b=10)

For b=10, the Pipkin diagrams in Fig. 2 show predominantly dark blue markers $\left(\Delta_{\text{RMS}}<0.1\right)$, indicating close agreement among the FENE, Cohen, and RS models across most of the (Wi,De) space. The limited regions of deviation highlight where the three force laws produce measurably different intracycle nonlinear responses. Overall, the broad agreement reflects two distinct limiting behaviors that dominate different parts of Pipkin space.

At low Weissenberg numbers (Wi≲1), all models reduce to their shared Hookean limit, equivalent to Oldroyd-B behavior [1,23]. Chain stretches remain small $\left(Q^{2}/b\ll 1\right)$, the viscous Lissajous–Bowditch curves are nearly elliptical, and higher harmonics such as $A_{3}/A_{1}$ are negligible [54,55]. Since the nonlinear curvature of the force law is not sampled in this regime, the three models coincide.

At the opposite extreme, in the large-strain-amplitude regime (upper-left, $\gamma_{0}>1$), the small extensibility parameter allows chains to approach their maximum extension during each oscillation cycle. As $Q\to \sqrt{b}(x\to 1)$, the spring force in all three models is dominated by the same ${\left(1-x^{2}\right)}^{-1}$ asymptotic behavior. In this saturation regime, geometric constraints overwhelm differences in the detailed curvature of the inverse Langevin approximations [27]. Although the Lissajous–Bowditch curves are strongly distorted, the intracycle dynamics become effectively model-independent, leading again to small $\Delta_{\text{RMS}}$ values.

Discrepancies ($\Delta_{\text{RMS}}≳0.2$, light green/yellow) are confined to a narrow band at high Deborah numbers (De≳25) and moderate Weissenberg numbers (5≲Wi≲15), corresponding to $\gamma_{0}\approx 0.2-0.6$, just below the linear–nonlinear transition diagonal. This region represents conditions where (i) chains are driven close to their extensibility limit (x≈0.8−1), and (ii) the oscillation period is short relative to the relaxation time, preventing full recoil between successive strain reversals. As a result, chains spend a significant fraction of each cycle in the strongly nonlinear regime, allowing differences in force-law curvature to accumulate dynamically.

This behavior is reflected in Fig. S2 (Supp. Material), where FENE systematically attains slightly smaller peak extensions than Cohen and RS. Asymptotic analysis in the supplemental material reveals that FENE’s dominant Jacobian eigenvalue grows more rapidly as x→1, indicating higher extension-dependent stiffness near the extensibility limit. Consequently, FENE resists full extension more strongly than the Cohen or RS models under comparable forcing.

We note that regions of large instantaneous extension also occur at higher strain amplitudes (upper-left of the Pipkin diagram), yet discrepancies remain small there because the response is dominated by universal saturation behavior. The transitional high—De, moderate—Wi band is unique in that chains are repeatedly driven near maximum extension while retaining sufficient dynamic sensitivity to differences in nonlinear curvature. In this regime, $A_{3}/A_{1}$ highlights the subtle differences among the models.

#### 3.1.2. Large extensibility limit (b=150)

For b=150, the Pipkin diagrams in Fig. 3 show a markedly different pattern of model agreement than in the b=10 case. The key difference arises from the larger maximum extension, $Q_{\text{max}}=\sqrt{b}\approx 12.2$, compared to $Q_{\text{max}}\approx 3.16$ for b=10. Because chains must undergo significantly greater deformation before approaching their extensibility limit, the saturation boundary is pushed to much larger strain amplitudes. This delays the onset of the universal ${\left(1-x^{2}\right)}^{-1}$ asymptotic behavior that renders all models equivalent, and consequently exposes a much broader region of Pipkin space to the transitional regime of moderate extension where the curvature differences among the three ILF approximations are most consequential.

As in the b=10 case, close agreement is recovered in the two limiting regimes: the Hookean limit at low Wi and the saturation limit at large strain amplitudes. For b=150, however, saturation requires much larger deformations, so the universal near-singular behavior is reached only at higher amplitudes. The gap between the linear and fully saturated regimes is therefore substantially wider. As a result, the transitional region where curvature differences among the ILF approximations are dynamically relevant expands across a broad swath of Pipkin space, spanning 5≲De≲50 and 5≲Wi≲15, and straddling the $\gamma_{0}=1$ diagonal rather than remaining confined below it as in the b=10 case.

### 3.2. Steady uniaxial extension

Uniaxial extensional flow imposes a constant elongational strain rate ${\dot{\epsilon }}_{0}$ along the primary x-axis, accompanied by equal contraction along the transverse directions. The dimensional velocity field is given by

(28) $$\tilde{u}=\left[{\dot{\epsilon }}_{0}\tilde{x},-\frac{1}{2}{\dot{\epsilon }}_{0}\tilde{y},-\frac{1}{2}{\dot{\epsilon }}_{0}\tilde{z}\right],$$

which, upon nondimensionalization with $\text{U}={\dot{\epsilon }}_{0}\text{L}$, yields the dimensionless velocity field

(29) $$u=\left[x,-\frac{1}{2}y,-\frac{1}{2}z\right].$$

The corresponding nondimensional stochastic evolution equation for the dumbbell configuration vector Q is

(30) $$dQ=(\nabla u{)}^{⊤}\cdot Qdt-\frac{1}{2De}F(Q)dt+\sqrt{\frac{1}{De}}dW_{t}.$$

Unlike LAOS, which introduces two independent time scales, steady uniaxial extension is time-independent and the flow strength is characterized by a single dimensionless parameter $De=Wi=\lambda {\dot{\epsilon }}_{0}$. This flow is a canonical test for finite chain extensibility because it drives polymer chains monotonically toward their maximum contour length, amplifying differences among ILF approximations that are suppressed in oscillatory flows. For Hookean dumbbells the extensional stress diverges at De=0.5; finite extensibility regularizes this singularity, and it is near this value that the extensional stress undergoes its most rapid growth with extension rate, making the coil-stretch transition the most sensitive region for model comparison.

#### 3.2.1. Transient and steady-state extensional viscosity

**Fig. 4.**
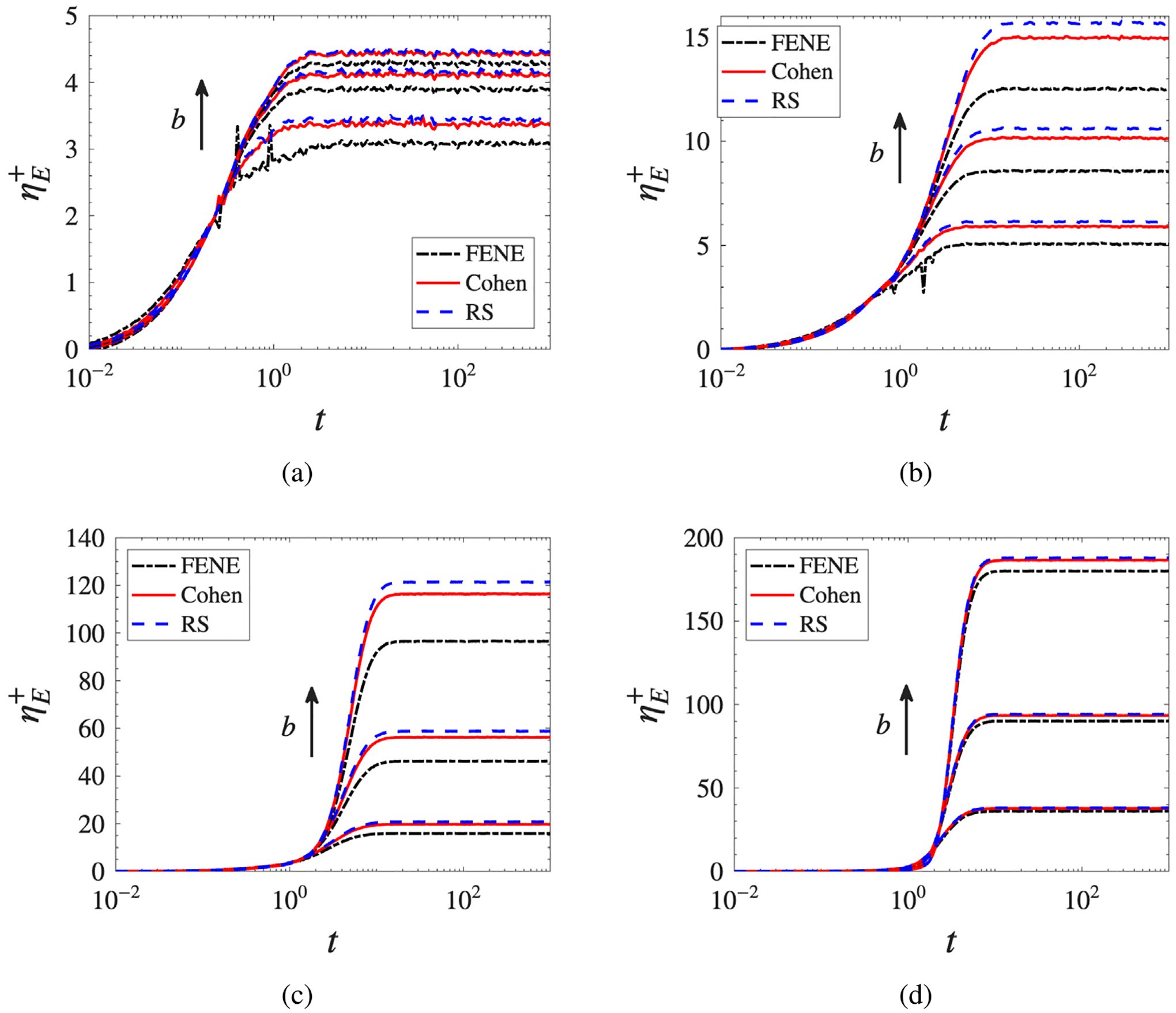
Transient extensional viscosity as a function of time for De=(a)0.25, (b) 0.5, (c) 1, (d) 5 and b=20,50,100, comparing the three ILF approximations.

Fig. 4 shows the transient extensional viscosity $\eta_{E}^{+}$ for extension rates spanning the weak-flow, transitional, and strong-flow regimes. At low extension rates (De=0.25), the flow is insufficient to fully uncoil the chains; the resulting viscosity enhancement is modest and the transient curves exhibit fluctuations characteristic of Brownian-dominated dynamics. Model differences are present but subtle, with FENE consistently predicting the lowest stress response. As the extension rate increases to De=0.5, near the coil-stretch transition, the strain-hardening response becomes pronounced and model differences grow appreciably, with FENE reaching a noticeably lower steady-state plateau than Cohen and RS. At De=1, the fluid exhibits fully developed strain-hardening: following an initial linear viscoelastic growth, the viscosity rises sharply as chains unravel and reaches a b-dependent plateau, with larger b supporting higher stress levels before full extension is reached. The FENE approximation consistently reaches a lower plateau than the Padé-based models, reflecting its lower predicted stress response. At high extension rates (De=5), the unfolding timescale is compressed and all models saturate rapidly; the qualitative ordering is preserved but the relative differences are compressed by the dominance of the hydrodynamic driving force.

**Fig. 5.**
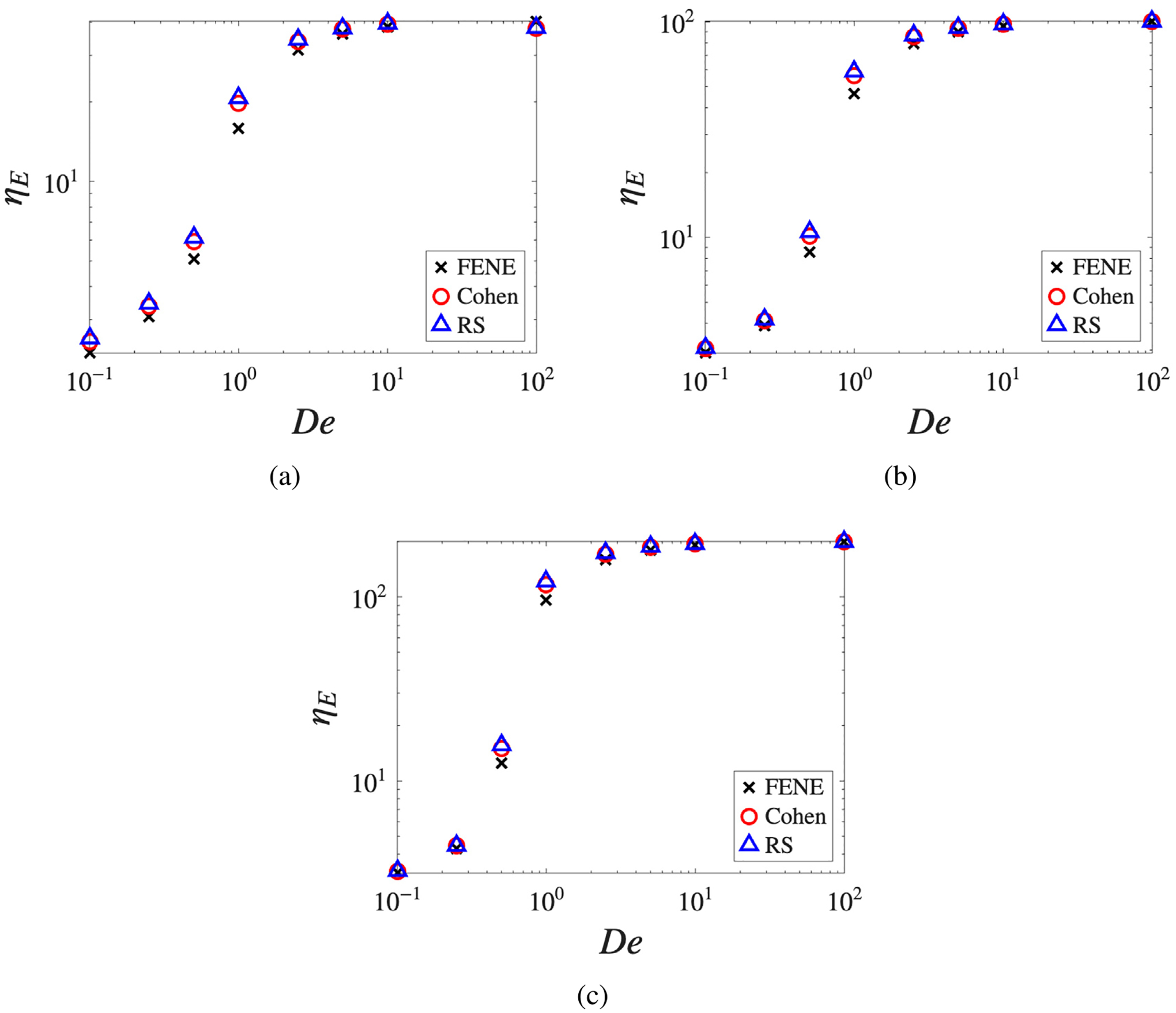
Steady-state extensional viscosity as a function of De for b=20,50,100, comparing the three ILF approximations.

The steady-state extensional viscosity as a function of De, shown in Fig. 5, further clarifies the regimes where model selection matters. For short chains (b=20), all three models collapse onto a single curve across the entire range of De: rapid saturation masks the subtle differences in spring-force curvature, consistent with the behavior observed in the b=10 LAOS results. As chain length increases to b=100, a clear divergence emerges in the transition region (De≈1−5), where FENE noticeably under-predicts the extensional viscosity relative to Cohen and RS. This confirms that for high-molecular-weight polymers the choice of ILF approximation plays a non-negligible role in predicting strain-hardening magnitude.

#### 3.2.2. Quantitative model comparison

**Fig. 6.**
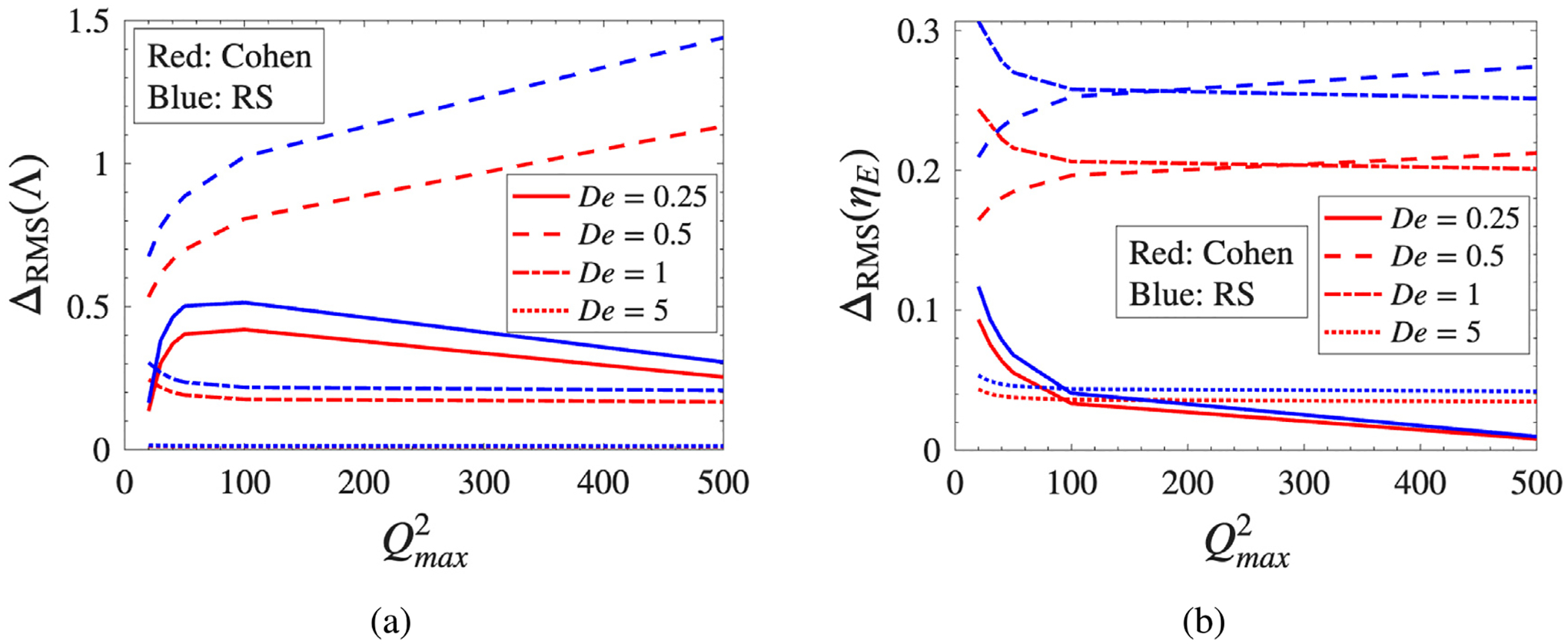
Normalized RMS difference, $\Delta_{\text{RMS}}$, of the Cohen and RS model predictions relative to FENE for (a) fractional stretch, Λ, and (b) extensional viscosity, $\eta_{E}$.

To quantify these deviations, we compute the normalized RMS difference of the steady-state extensional viscosity $\left(\eta_{E}=\left(\tau_{xx}-\tau_{yy}\right)/De\right)$ and fractional stretch $\left(\Lambda =\sqrt{\text{tr}\;\tau /3}/Q_{\text{max}}\right)$:

(31) $$\Delta_{\text{RMS}}([\cdot ])=\frac{1}{[\cdot {]}_{\text{FENE}}}\sqrt{\frac{1}{N}\sum_{i=1}^{N}{\left([\cdot {]}_{i,\text{model}}-[\cdot {]}_{i,\text{FENE}}\right)}^{2}},$$

where FENE serves as the reference. The results are shown in Fig. 6.

For the fractional stretch (Fig. 6a), deviations are largest near the coil-stretch transition. At De=0.25, the RS model exhibits differences exceeding 50% for b≈100, with Cohen reaching approximately 40%; these reflect the sensitivity of the equilibrium stretch prediction to spring-law curvature in the weak-flow regime. Deviations grow further at De=0.5, where the RS model approaches 150% difference at high extensibility, marking this as the most sensitive operating point for model selection. At high extension rates (De=1 and De=5), differences collapse to below 5% as hydrodynamic drag dominates and all models are driven toward the same fully stretched asymptote.

The viscosity deviations (Fig. 6b) follow the same qualitative trends but with an important distinction: unlike the stretch metric, the viscosity differences do not fully collapse at De=1, stabilizing instead around 20% for both Cohen and RS. This residual offset persists because, even when models predict similar chain extensions, their stress–conformation relationships differ, leading to distinct stress predictions at the same kinematic state. Convergence is only achieved at De=5, consistent with full chain saturation. The persistent offset at De≈0.5 underscores that ILF curvature differences influence macroscopic stress predictions independently of whether the chains have reached their extensibility limit.

#### 3.2.3. Microscopic origins: configuration distributions

**Fig. 7.**
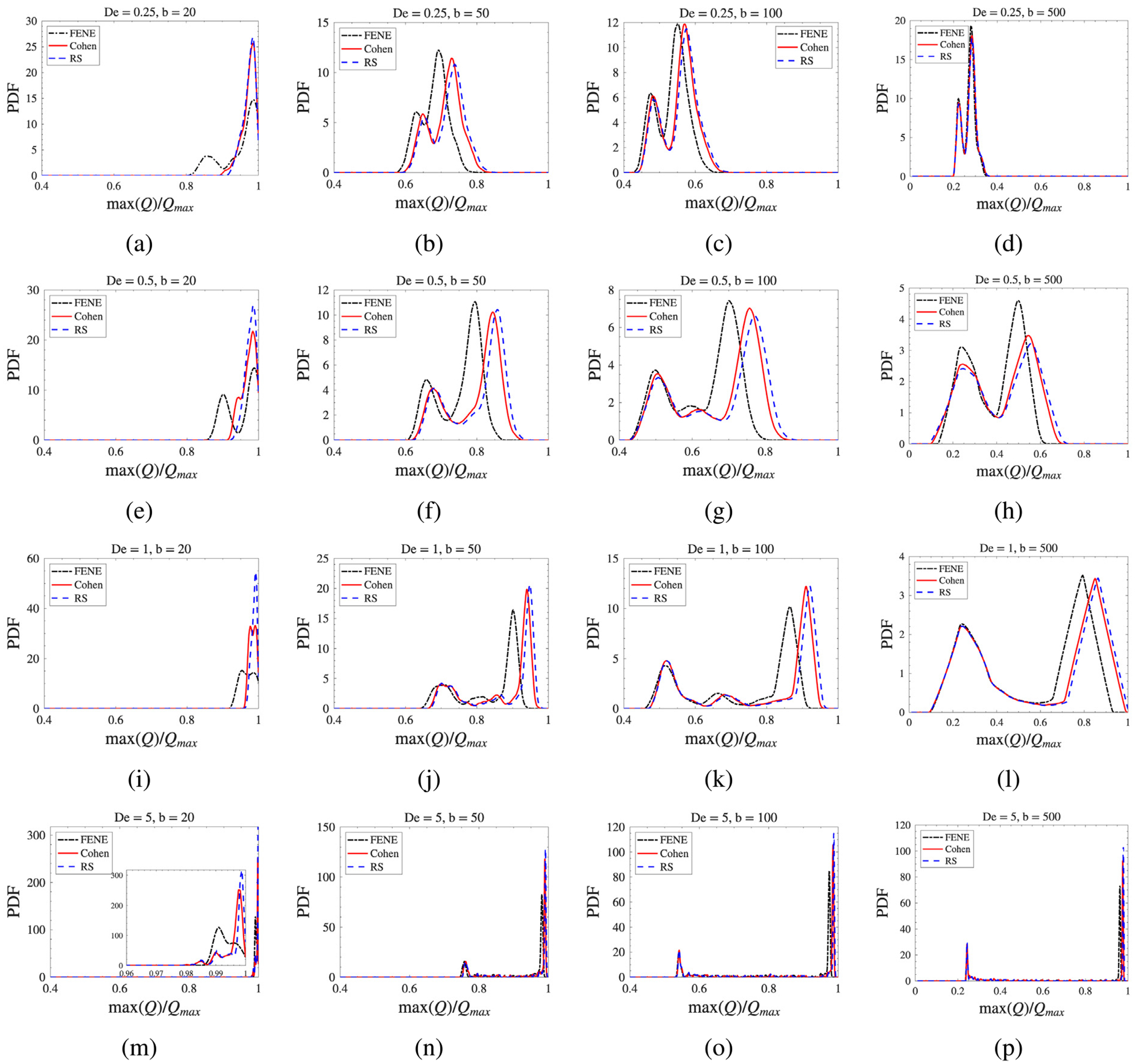
Probability density functions (PDFs) of the normalized maximum dumbbell extension, $\text{max}(Q)/Q_{\text{max}}$, comparing the three ILF approximations. Rows correspond to De=0.25,0.5,1,5; columns correspond to b=20,50,100,500.

The macroscopic differences described above are rooted in the microscopic configuration distributions of the dumbbell ensemble. Fig. 7 shows the probability density functions (PDFs) of the normalized maximum extension $\text{max}(Q)/Q_{\text{max}}$ across the (De,b) parameter space.

The influence of De follows a clear trend: increasing the extension rate shifts the ensemble from a predominantly coiled state at De=0.25 to a highly stretched state at De=5, with the intermediate regime (De=0.5−1) exhibiting significant multimodality as the ensemble splits between coiled and stretched populations near the coil-stretch transition. The role of b is equally important: as b decreases from 500 to 20, the accessible configuration space narrows dramatically, producing nearly singular probability peaks at b=20 with intensities an order of magnitude higher than those at b=100. Shorter chains therefore have a much narrower window of accessible states between the equilibrium coil and the fully stretched limit, which is why model differences are suppressed at small b.

Within these regimes, the three models display a consistent hierarchy. FENE consistently predicts peaks at lower extension values, confirming that it predicts the lowest ensemble-averaged stretch at moderate extensions—even at high De it retains a more relaxed ensemble configuration than Cohen and RS. The Padé-based models predict higher ensemble-averaged stretch, with RS frequently exhibiting the highest probability of near-maximum extension. This hierarchy is most clearly exposed at large b, where the wider configuration space prevents saturation from masking the spring-law differences, and is most pronounced near the coil-stretch transition (De≈0.5−1).

#### 3.2.4. Numerical stiffness near full extension

At high De, as chains approach full extension $\left(Q^{2}\to b\right)$, the governing equations become increasingly stiff due to the singular behavior of the spring force. As shown in the supplemental material, linearization of the deterministic dynamics reveals that the dominant eigenvalue $\lambda_{+}$ diverges as ${\left(b-Q^{2}\right)}^{-2}$, with a model-dependent prefactor. FENE exhibits the largest prefactor (equal to 1), while Cohen and RS are 33–40% less stiff, consistent with their smaller eigenvalue growth rates and closer approximation to the exact ILF near $x\to 1^{-}$.

Although this stiffness becomes significant near full extension, it does not pose major challenges in viscometric flows, where the spatially uniform and temporally constant velocity gradient allows the microstructure equations (Eq. (8a)) to be solved independently of the macroscopic flow. The semi-implicit scheme described in the supplemental material handles the eigenvalue growth effectively by treating the nonlinear force term implicitly through the scalar cubic equation (Eq. S9). However, in non-viscometric flows where spatial velocity gradients couple the microstructure dynamics to transient macroscopic evolution, this stiffness can compromise solver stability in ways that depend on the choice of ILF approximation, as discussed in Section 4.

## 4. Non-viscometric flow: Capillary thinning

Capillary thinning provides a natural non-viscometric counterpart to the steady uniaxial extension results of Section 3. In capillary-driven filament thinning, the local flow in the necked region is predominantly extensional, so the dynamics are closely linked to the underlying extensional constitutive response. Prior studies have shown that capillary thinning and filament stretching provide complementary measures of extensional rheology, with the apparent extensional viscosity inferred from thinning approaching the same steady-state plateau measured in filament stretching [56]. In this framework, the elastocapillary regime is associated with the buildup of extensional stress and a strain-dependent growth of the transient extensional viscosity, whereas the terminal visco-elastocapillary regime reflects finite extensibility and the approach to a terminal extensional viscosity [57]. Capillary-thinning dynamics are also influenced by coil-stretch hysteresis and conformation-dependent polymer friction, including stretching-induced self-concentration/self-dilution effects [58]. This connection motivates the inclusion of capillary thinning here: the steady extensional results identify the moderate-extension/strain-hardening regime as the region where the three ILF approximations differ most strongly, and the capillary-thinning problem allows us to examine how those same constitutive differences manifest in a coupled transient flow. Unlike the viscometric calculations of Section 3, where the microstructural equations decouple from the conservation laws, capillary thinning requires full micro–macro coupling and therefore provides a more stringent test of the numerical consequences of the ILF choice. The results below confirm that the largest model differences are associated with the moderate-extension/strain-hardening regime identified in steady extension, while also showing how those constitutive differences feed into numerical stability and attainable simulation time in a fully coupled flow.

To examine these effects in a coupled non-viscometric setting, we consider an axisymmetric slender jet of polymeric liquid thinning due to surface tension. The radius of the jet slowly varies along the liquid jet and we only consider the leading-order approximation in an expansion in the radius [59,60]. The conservation of volume and momentum along the jet can be written as follows

(32) $$\frac{\partial {\tilde{R}}^{2}}{\partial \tilde{t}}+\frac{\partial }{\partial \tilde{z}}\left(\tilde{u}{\tilde{R}}^{2}\right)=0$$

(33) $$\rho \left(\frac{\partial \tilde{u}}{\partial \tilde{t}}+\tilde{u}\frac{\partial \tilde{u}}{\partial \tilde{z}}\right)=-\gamma \frac{\partial \tilde{\kappa }}{\partial \tilde{z}}+3\eta_{s}\frac{1}{{\tilde{R}}^{2}}\frac{\partial }{\partial \tilde{z}}\left({\tilde{R}}^{2}\frac{\partial \tilde{u}}{\partial \tilde{z}}\right)+\frac{1}{{\tilde{R}}^{2}}\frac{\partial }{\partial \tilde{z}}\left({\tilde{R}}^{2}{\tilde{\sigma }}_{E}\right),$$

where ρ is the density, γ is the surface tension, $\kappa =\frac{1}{R{\left(1+R_{z}^{2}\right)}^{1/2}}-\frac{R_{zz}}{{\left(1+R_{z}^{2}\right)}^{3/2}}$ is the curvature, and $\eta_{S}$ is the solvent viscosity. The polymer contribution is given by the tensile stress $\sigma_{E}=\tau_{zz}-\tau_{rr}$, obtained from Eq.(6).

Scaling using the relations given in Eq.(3), with characteristic length $L=R_{0}$, where $R_{0}$ is the initial radius of the jet and the time scale given by the Rayleigh time scale, such that $t_{R}=U/L=\sqrt{\rho R_{0}^{3}/\gamma }$, we obtain

(34) $$\frac{\partial R^{2}}{\partial t}+\frac{\partial }{\partial z}\left(uR^{2}\right)=0$$

(35) $$\frac{\partial u}{\partial t}+u\frac{\partial u}{\partial z}=-\frac{\partial \kappa }{\partial z}+\frac{3\beta Oh}{R^{2}}\frac{\partial }{\partial z}\left(R^{2}\frac{\partial u}{\partial z}\right)+\frac{\left(1-\beta \right)Oh}{De}\frac{1}{R^{2}}\frac{\partial }{\partial z}\left(R^{2}\left(\tau_{zz}-\tau_{rr}\right)\right),$$

where the Deborah number is defined as before, so that $De=\lambda \frac{U}{L}=\lambda \sqrt{\gamma /\rho R_{0}^{3}}=\lambda /t_{R}$. And the Ohnesorge number is $Oh=\eta_{0}/\sqrt{\rho \gamma R_{0}}$, where $\eta_{0}=\eta_{s}+\eta_{p}$.

### 4.1. Macroscopic numerical solver

The axisymmetric macroscopic Eqs. (34)–(35), with the polymeric extra-stress components $\tau_{zz}$ and $\tau_{rr}$ provided by the microscopic model, are solved using a finite-difference method [61]. All spatial derivatives, except for the convective terms, are discretized using second-order central differences.

For convective terms of the form $u\partial_{z}f$, a velocity-weighted blend of central and upwind differencing is employed. This approach maintains numerical stability at high local flow speeds while preserving second-order accuracy in regions of slow flow. Specifically, the first derivative in the axial direction at grid point j is approximated as

(36) $${\left(\frac{\partial f}{\partial z}\right)}_{j}=\frac{1-g_{j}}{2\Delta z}\left(f_{j+1}-f_{j-1}\right)+\frac{g_{j}}{\Delta z}\left\{\begin{array}{ll} f_{j}-f_{j-1}, & u_{j}\geq 0,\\ f_{j+1}-f_{j}, & u_{j}<0, \end{array}\right.$$

where the weighting parameter is defined as $g_{j}=\left|u_{j}\right|/u_{\text{max}}$ and $u_{\text{max}}$ is a reference velocity, typically chosen as the current maximum axial velocity in the computational domain. In the limit $g_{j}\approx 0$, the scheme reduces to pure second-order central differencing, while for $g_{j}\approx 1$ it approaches a first-order upwind scheme aligned with the local flow direction.

Time integration is performed using a first-order implicit Euler method. Second-order temporal accuracy is recovered through a time-filtering procedure [62], which preserves the unconditional stability of the underlying scheme. Variable time stepping is implemented via an embedded error estimator, significantly accelerating the simulations, particularly during the slow initial stages of filament evolution.

The computational domain spans one half-wavelength of the imposed perturbation, 0≤z≤π/k. At the domain boundaries, z=0 and z=π/k, symmetry (reflection) boundary conditions corresponding to a half-wavelength perturbation are applied, namely u=0 and $\partial_{z}R=0$.

The velocity field is initially set to zero, and the polymer configuration is initialized in its equilibrium state. The filament radius is prescribed as a small-amplitude sinusoidal perturbation of the uniform radius $R_{0}$,

(37) $$R\left(z,0\right)=R_{0}\left(1+0.01\;\text{cos}\left(kz\right)\right),$$

following Ardekani et al. [63].

At each macroscopic time step, the coupled nonlinear system governing the axial velocity and filament radius is solved using a Newton–Raphson iteration with an analytical Jacobian. After solving the macroscopic equations, the updated velocity field is used to compute the velocity-gradient tensor ∇u at every grid point. These gradients, together with the local velocity, are passed to the microscopic solver, which advances the dumbbell configurations. The resulting polymeric stress contributions are returned via Eq. (8b) and used in the subsequent macroscopic time step.

### 4.2. Spatial resolution and ensemble size

**Fig. 8.**
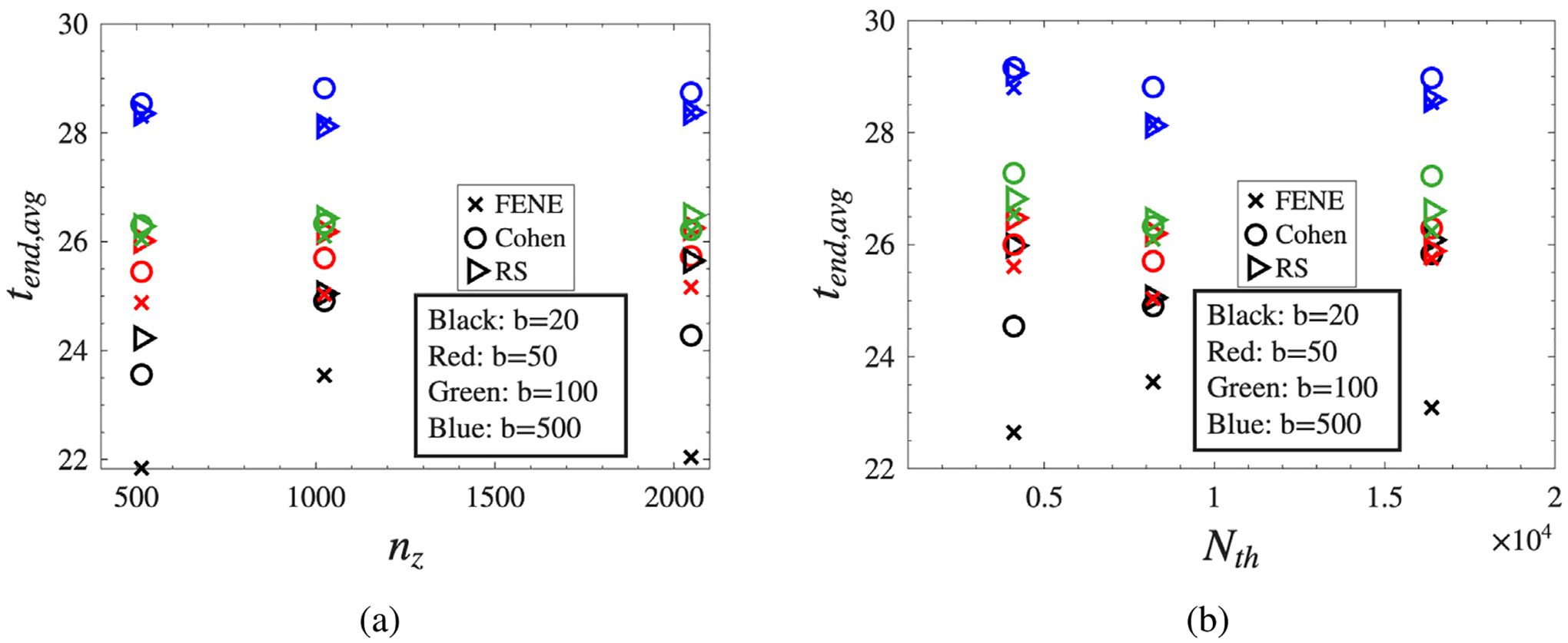
Average final simulation time $t_{\text{end,avg}}$ as a function of (a) spatial resolution $n_{z}$ and (b) ensemble size $N_{\text{th}}$. Results are shown for three ILF approximations: FENE (×), Cohen (◦), and RS (⊳), with finite extensibility parameters b=20 (black), b=50 (red), b=100 (green), and b=500 (blue). Averages are computed over five independent simulations using fixed random seeds, with fixed parameters: viscosity ratio β=0.27, Ohnesorge number Oh=0.4, Deborah number De=2, and wavelength k=0.8. The weak dependence on $n_{z}$ and $N_{\text{th}}$ relative to the systematic separation across b indicates that the reported trends are not controlled by spatial or ensemble resolution.

To assess the robustness of our numerical solver, we evaluated its sensitivity to spatial resolution $\left(n_{z}\right)$ and the total number of configuration fields (dumbbells) per spatial grid point $\left(N_{\text{th}}\right)$. For this analysis, we performed five simulations per configuration, each using distinct but fixed random seeds to account for stochastic effects. We consider four extensibility parameters, b=20,50,100,500, so that the numerical robustness can be assessed across a broad range of chain extensibilities. Fig. 8 illustrates the average final simulation time $t_{\text{end,avg}}$, computed across these five runs, as a function of resolution for all three ILF approximations (FENE, Cohen, and RS) and the four finite extensibility parameters (b=20,50,100,500), while holding the viscosity ratio β=0.27, Ohnesorge number Oh=0.4, Deborah number De=2, and wavelength k=0.8 constant. Because $t_{\text{end,avg}}$ is determined by adaptive time stepping in a stochastic simulation, its dependence on $n_{z}$ and $N_{\text{th}}$ is not expected to be strictly monotonic. The purpose of Fig. 8 is therefore to verify that the observed trends are only weakly sensitive to spatial and ensemble resolution once these are sufficiently large.

In panel (a), as spatial resolution $n_{z}$ increases from 500 to 2000, $t_{\text{end,avg}}$ changes only modestly relative to the systematic separation across b. The FENE approximation (× symbols) shows slightly lower values compared to Cohen (∘) and RS (⊳) in several cases, but the dominant trend is the increase in $t_{\text{end,avg}}$ with increasing b. More generally, $t_{\text{end,avg}}$ tends to be smaller for low-extensibility cases and larger for high-extensibility cases. The spread between ILF approximations is most apparent for b=20 and becomes smaller for larger b, consistent with the approach to Hookean behavior as b→∞.

Panel (b) presents analogous results versus the number of dumbbells $N_{\text{th}}$, ranging from 0.5×10^4^ to 2×10^4^. A similar picture emerges: once $N_{\text{th}}$ is of order 10^4^, further increases produce only modest changes in $t_{\text{end,avg}}$ relative to the systematic shifts associated with b and, to a lesser extent, the ILF approximation. Overall, these tests confirm that the solver is not unduly sensitive to moderate changes in spatial or ensemble resolution, validating its use for the parameter regimes explored in this study.

### 4.3. Effect of ILF approximations on chain-stretch statistics and numerical performance

To characterize the interplay between microscopic dynamics and numerical performance in capillary thinning, we track two complementary metrics throughout each simulation. The first is the fraction of dumbbells satisfying $Q/Q_{\text{max}}>0.94$, denoted $P\left(Q/Q_{\text{max}}>0.94\right)$. At each saved time step, this quantity is computed as the ratio of chains whose instantaneous extension exceeds 94% of the maximum contour length to the total number of dumbbells across all spatial grid points. It therefore measures the evolution of the highly stretched tail of the configuration distribution and is sensitive to differences among spring laws near full extension.

The second metric is the final simulation time $t_{\text{end}}$, defined as the last time at which the adaptive time-stepping scheme could advance the solution before the macro time step fell below the threshold $\Delta t<10^{-8}$, at which point the computation is terminated. As discussed in the supplemental material, the numerical stiffness of the coupled micro–macro system grows sharply as configurations approach full extension, and the prefactor governing this divergence is ILF-dependent. Accordingly, $t_{\text{end}}$ serves as a numerical diagnostic of solver robustness for each spring law rather than a physical breakup time.

**Fig. 9.**
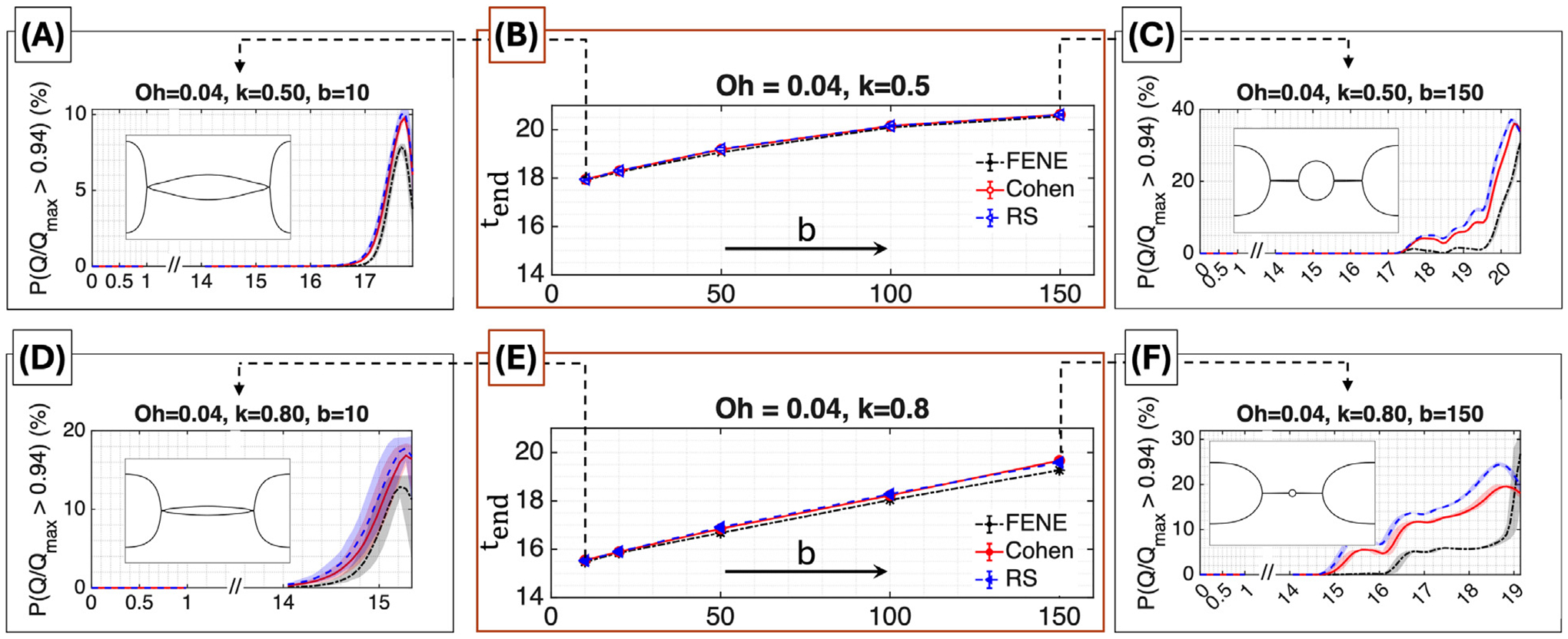
Final simulation time and microscopic stretch statistics at Oh=0.04. *Top row* (k=0.5): Time evolution of $P\left(Q/Q_{\text{max}}>0.94\right)$ for b=10 (A) and b=150 (C), with shaded min–max bands across seeds. Numerical termination time $t_{\text{end}}$ versus b (B). *Bottom row* (k=0.8): Time evolution of $P\left(Q/Q_{\text{max}}>0.94\right)$ for b=10 (D) and b=150 (F). $t_{\text{end}}$ versus b (E). Snapshots show final filament shapes.

Fig. 9 presents results for Oh=0.04. In this low-Ohnesorge regime, inertial stresses are comparable to viscous stresses, leading to rapid and strongly inertia-dominated filament thinning, as typically observed in inkjet drop formation or spray atomization of dilute polymer solutions. The middle panels, (B) and (E), show $t_{\text{end}}$ versus the extensibility parameter b for wavelengths k=0.5 and k=0.8, respectively. In both cases, the FENE, Cohen, and RS curves are nearly indistinguishable across b, indicating that in this fast-thinning regime the specific ILF approximation has limited influence on numerical stability.

Panels (A), (C), (D), and (F) show the time evolution of $P\left(Q/Q_{\text{max}}>\right.0.94)$, with shaded bands indicating variability across random seeds. Differences among the ILF approximations are evident here, particularly at larger b. At intermediate times, FENE generally yields lower values of $P\left(Q/Q_{\text{max}}>0.94\right)$ than the Padé-based models. This trend is consistent with the fact that at a given moderate extension, the FENE spring law provides a stronger restoring force, which locally resists further thinning and reduces the instantaneous strain rate, thereby limiting the fraction of highly stretched chains. The Cohen and RS models, having a weaker force-extension response over this moderate-extension range, allow faster stretching and therefore larger values of $P\left(Q/Q_{\text{max}}>0.94\right)$.

The wavenumber also influences the response. For k=0.5 and b=10 [panel Fig. 9(A)], $P\left(Q/Q_{\text{max}}>0.94\right)$ rises sharply as necking develops and subsequently decreases as the strain rate weakens and the chains relax. At b=150 [panel Fig. 9(C)], both the Cohen and RS models exhibit a peak followed by a decline before the simulation ends. In contrast, the FENE model increases monotonically throughout the simulated window and does not display a peak-and-decay behavior. This difference is consistent with the earlier discussion of the models’ force-extension characteristics (see the paragraph above on moderate-extension behavior), which lead to distinct short- and intermediate-time stretching dynamics. For k=0.8, the shorter wavelength confines more mass near the domain endpoints and yields a thinner mid-filament and a smaller satellite. Consequently, $P\left(Q/Q_{\text{max}}>0.94\right)$ evolves more gradually and may exhibit oscillatory behavior associated with repeated localized stretching and partial relaxation [panels Fig. 9(D,F)]. At large b and k=0.8 [panel Fig. 9(F)], a late-time inversion occurs in which the FENE curve overtakes the Padé models shortly before termination. This reflects the accumulation of near-limit configurations under FENE and its larger near-limit numerical stiffness, which accelerates the collapse of the adaptive time step and leads to the slightly lower $t_{\text{end}}$ observed in panel (E).

**Fig. 10.**
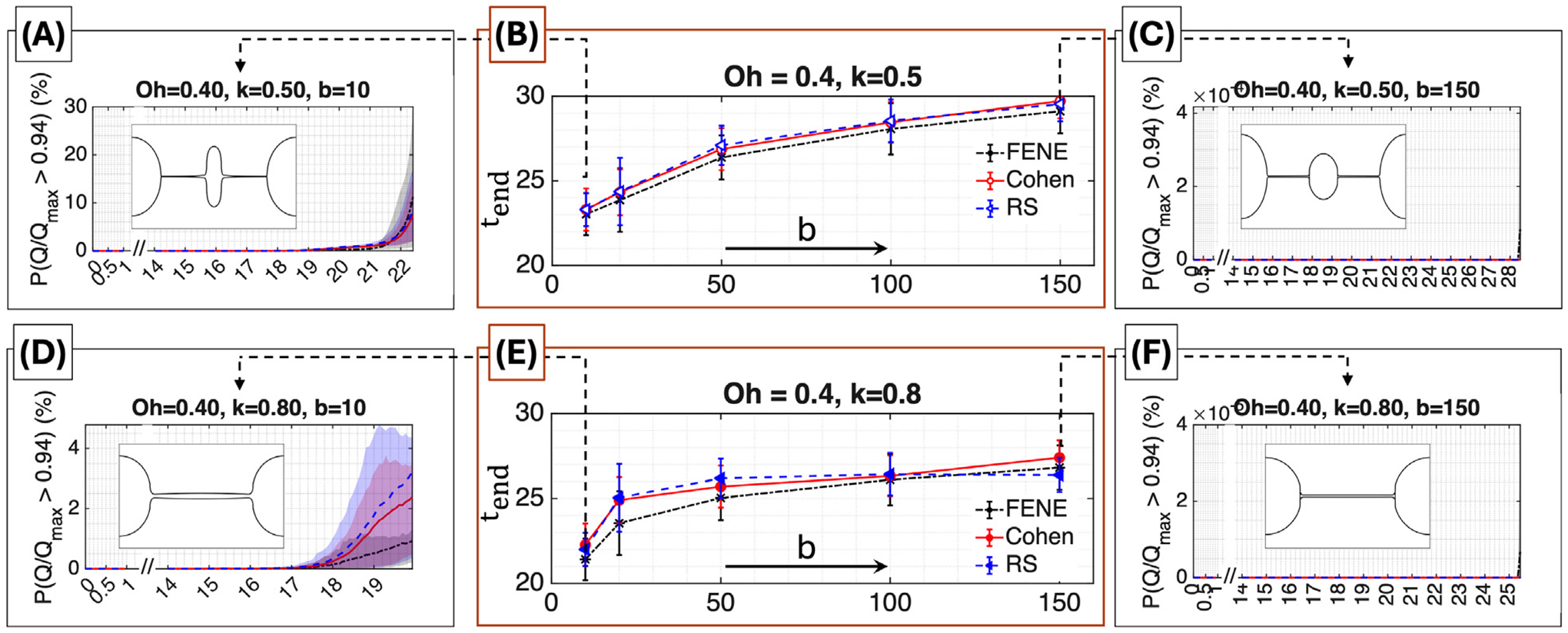
Final simulation time and microscopic stretch statistics at Oh=0.4. *Top row* (k=0.5): Time evolution of $P\left(Q/Q_{\text{max}}>0.94\right)$ for b=10 (A) and b=150 (C), with shaded min–max bands across seeds. Numerical termination time $t_{\text{end}}$ versus b (B). *Bottom row* (k=0.8): Time evolution of $P\left(Q/Q_{\text{max}}>0.94\right)$ for b=10 (D) and b=150 (F). $t_{\text{end}}$ versus b (E). Snapshots show final filament shapes.

Fig. 10 shows the corresponding results for Oh=0.4, a high-Ohnesorge regime in which viscous forces dominate and thinning is slow, as encountered in CaBER experiments, highly viscous jet stabilization, and embedded-filament extrusion. In contrast to the Oh=0.04 case, the slower dynamics substantially increase sensitivity to the ILF approximation. In panels (B) and (E), $t_{\text{end}}$ for FENE is consistently lower than for Cohen and RS across all values of b and for both wavenumbers. This arises from two factors: (i) at moderate stretches, FENE provides stronger elastic resistance at a given extension, leading to stronger feedback into the macroscopic evolution and more stringent time-step restrictions; and (ii) near full extension, FENE exhibits a higher numerical stiffness prefactor (50%–67% larger than Cohen or RS—see Supp. Material), which further reduces the attainable final time step.

Panels (A), (C), (D), and (F) show the evolution of $P\left(Q/Q_{\text{max}}>0.94\right)$ with shaded seed variability. In particular, panel Fig. 10(D) shows high variability with FENE consistently yielding smaller $P\left(Q/Q_{\text{max}}>0.94\right)$ than Cohen and RS, reflecting its stronger resistance to extensional deformation at moderate stretch. The broader seed variability arises because the slow viscous dynamics allow stochastic fluctuations to accumulate over long timescales, in contrast to the rapid deterministic driving observed at Oh=0.04. Finally, for large b=150 [panels Fig. 10(C,F)], $P\left(Q/Q_{\text{max}}>0.94\right)$ remains near zero for all closures. In these cases, few dumbbells reach the near-maximum extension regime, and termination results from the formation of sharp macroscopic gradients as the filament approaches pinch-off rather than from microstructural stiffness. This distinction highlights that $t_{\text{end}}$ can reflect either *numerical stiffness arising from near*-*limit chain configurations* when chains approach full extension, or *macroscopic geometric singularity* when the filament shape becomes ill-conditioned, depending on the flow regime.

## 5. Conclusions

We examined how the mathematical representation of finite extensibility — via approximations to the inverse Langevin function (ILF) — influences stochastic dumbbell dynamics when the *full* microstructural equations are solved without constitutive closure. Within the Brownian Configuration Field (BCF) framework, we compared three widely used spring laws — the classical FENE model and the Padé-type approximations of Cohen and Rickaby–Scott — across viscometric (LAOS, steady uniaxial extension) and non-viscometric (capillary thinning) flows, thereby isolating how the spring law shapes polymer stretching, stress evolution, and numerical robustness in coupled micro–macro simulations.

In viscometric flows, all three ILF approximations show agreement in the two limiting regimes of deformation: (i) weak forcing, where chains remain Hookean, and (ii) strongly driven conditions where chains rapidly approach their extensibility limit. By contrast, *transitional* regimes with *moderate extension* reveal systematic, closure-dependent differences: FENE predicts lower ensemble-averaged stretch and stresses than the Padé models, with discrepancies that grow with the extensibility parameter b. These differences appear in both time-dependent (LAOS harmonics) and steady (strain-hardening) responses, demonstrating that spring-law curvature at moderate extension alters macroscopic predictions well before saturation.

In LAOS, the role of the extensibility parameter b is manifested through the width of the transitional regime in which curvature differences among the ILF approximations remain dynamically relevant. For small b, chains approach their extensibility limit readily, and rapid saturation suppresses closure-dependent differences across much of Pipkin space. Observable discrepancies are therefore confined to a narrow band at high Deborah number and moderate strain amplitude, where chains attain large but not fully saturated extensions and remain sensitive to differences in spring-force curvature. In contrast, increasing b delays the onset of saturation, preventing premature masking of curvature effects and broadening the region of parameter space over which ILF-dependent differences influence the nonlinear harmonic response.

In steady uniaxial extension, the influence of the ILF approximation is even more pronounced, particularly near the coil-stretch transition (De≈0.5−1). While all models coincide in the weak-flow limit and converge again under strongly driven conditions where chains saturate, significant quantitative differences emerge in the transitional regime. FENE systematically predicts lower steady-state stretch and extensional viscosity than the Padé closures, with deviations that increase with extensibility parameter b. For highly extensible chains, normalized RMS differences in fractional stretch and viscosity become largest near the coil-stretch transition, indicating that spring-law curvature directly alters the balance between hydrodynamic stretching and entropic recoil before full extension is reached. Notably, viscosity discrepancies persist even when predicted stretches are similar, reflecting differences in stress–conformation coupling intrinsic to the ILF approximation. These results demonstrate that in extensional flows — where chains are monotonically driven toward large extension — the choice of inverse Langevin representation can materially influence strain-hardening predictions well before the extensibility limit is attained.

For capillary thinning, the impact of the ILF is regime dependent. At low Ohnesorge number (Oh=0.04), representative of fast, inertiainfluenced thinning such as drop-on-demand inkjet breakup and spray atomization, the three closures yield *closely clustered* numerical termination times across the range of b considered, even though the near-limit stretch fraction $P\left(Q/Q_{\text{max}}>0.94\right)$ exhibits clear model-dependent differences throughout the thinning process, reflecting genuine physical disagreement among the spring laws at the molecular level. At higher Ohnesorge number (Oh=0.4), characteristic of slowly evolving viscous thinning (e.g., CaBER, highly viscous jet stabilization, embedded filament extrusion), systematic differences emerge and *increase with* b. Longer dwell times at large stretch amplify small curvature differences between ILFs into measurable shifts in stress amplification and in numerical termination time, indicating that the ILF choice can become quantitatively important when polymers more fully explore the nonlinear stretch regime.

The ILF choice also controls numerical stiffness in the coupled solver. Linearization near full extension shows that the dominant eigenvalue grows as ${\left(b-Q^{2}\right)}^{-2}$ with a model-dependent prefactor; FENE has the largest prefactor, implying 𝒪(50−67%) greater numerical stiffness than the Padé models and a need for correspondingly smaller time steps near the extensibility limit. Consistent with this analysis, capillary-thinning runs exhibit earlier adaptive-step collapse (smaller $t_{\text{end}}$) under FENE in regimes where the near-limit ensemble fraction is appreciable. In cases where that fraction remains negligible — as occurs for large b at Oh=0.4, where extensional rates are insufficient to drive a significant population of chains beyond $Q/Q_{\text{max}}>0.94$ — termination is instead governed by macroscopic geometric gradients as the filament approaches pinch-off, rendering the finite-difference discretization increasingly ill-conditioned independently of the microstructural dynamics.

Taken together, these results demonstrate that the inverse Langevin approximation plays a substantive role in stochastic dumbbell simulations rather than serving as a secondary implementation detail. Differences among approximations arise systematically in transitional regimes where chains experience moderate extension, influencing strain-hardening predictions in viscometric flows well before full saturation is reached. In strongly coupled non-viscometric flows, the ILF choice further affects numerical stiffness and attainable simulation time through model-dependent eigenvalue growth near the extensibility limit. The Padé-based approximations examined here exhibit reduced numerical stiffness relative to FENE and more closely track the exact inverse Langevin function in the moderate-extension regime explored in this study, especially for highly extensible chains. Accordingly, the selection of inverse Langevin representation should be regarded as a deliberate modeling decision when predictive accuracy and computational stability are both essential.

## Supplementary Material

Supp Material

## Data Availability

Data will be made available on request.

## Appendix A. Supplementary data

Supplementary material related to this article can be found online at https://doi.org/10.1016/j.jnnfm.2026.105610.

## References

[1] Bird RB, Curtiss CF, Armstrong RC, Hassager O, Dynamics of polymeric liquids, in: Kinetic Theory, vol. 2, Wiley, 1987.

[2] Kröger M, Simple models for complex nonequilibrium fluids, Phys. Rep 390 (6) (2004) 453–551.

[3] Hulsen M, Van Heel A, Van Den Brule B, Simulation of viscoelastic flows using Brownian configuration fields, J. Non-Newton. Fluid Mech 70 (1–2) (1997) 79–101.

[4] Larson RG, The structure and rheology of complex fluids, in: Topics in Chemical Engineering, first ed. Oxford University Press, New York, 1999.

[5] Rubinstein M, Colby RH, Polymer Physics, Oxford University Press, New York, 2003.

[6] Ghosh I, McKinley GH, Brown RA, Armstrong RC, Deficiencies of FENE dumbbell models in describing the rapid stretching of dilute polymer solutions, J. Rheol 45 (3) (2001) 721–758.

[7] Haward SJ, Oliveira MS, Alves MA, McKinley GH, Optimized cross-slot flow geometry for microfluidic extensional rheometry, Phys. Rev. Lett 109 (12) (2012) 128301. 10.1103/PhysRevLett.109.128301 23005994

[8] Haward SJ, McKinley GH, Shen AQ, Elastic instabilities in planar elongational flow of monodisperse polymer solutions, Sci. Rep 6 (1) (2016) 33029. 27616181 10.1038/srep33029 PMC5018825

[9] Dinic J, Sharma V, Flexibility, extensibility, and ratio of Kuhn length to packing length govern the pinching dynamics, coil-stretch transition, and rheology of polymer solutions, Macromolecules 53 (12) (2020) 4821–4835.

[10] Clasen C, Eggers MA, Fontelos J, Li J, McKinley GH, The beads-on-string structure of viscoelastic threads, J. Fluid Mech 556 (2006) 283–308.

[11] Oliveira MS, Yeh R, McKinley GH, Iterated stretching, extensional rheology and formation of beads-on-a-string structures in polymer solutions, J. Non-Newton. Fluid Mech 137 (1–3) (2006) 137–148.

[12] Calabrese V, Shen AQ, Haward SJ, How do polymers stretch in capillary-driven extensional flows? Macromolecules 57 (20) (2024) 9668–9676.

[13] Khalid M, Badoni A, Dutta D, Naidu P, Subramanian G, Shankar V, Role of finite extensibility on the centre-mode instability in viscoelastic channel flow, J. Fluid Mech 1009 (2025) A28.

[14] Zhang M, Lashgari I, Zaki TA, Brandt L, Linear stability analysis of channel flow of viscoelastic Oldroyd-B and FENE-P fluids, J. Fluid Mech 737 (2013) 249–279.

[15] Beneitez M, Page J, Kerswell RR, Polymer diffusive instability leading to elastic turbulence in plane Couette flow, Phys. Rev. Fluids 8 (10) (2023) L101901.

[16] Yamani S, McKinley GH, Master curves for FENE-P fluids in steady shear flow, J. Non-Newton. Fluid Mech 313 (2023) 104944.

[17] Zinelis K, Abadie T, McKinley GH, Matar OK, The fluid dynamics of a viscoelastic fluid dripping onto a substrate, Soft Matter 20 (41) (2024) 8198–8214. 39365107 10.1039/d4sm00406j

[18] Zinelis K, Abadie T, McKinley GH, Matar OK, Transition to elasto-capillary thinning dynamics in viscoelastic jets, J. Fluid Mech 998 (2024) A4.

[19] Somasi M, Khomami B, Woo NJ, Hur JS, Shaqfeh ESG, Brownian dynamics simulations of bead–rod and bead–spring chains: numerical algorithms and coarse–graining issues, J. Non-Newton. Fluid Mech 108 (2002) 227–255.

[20] Underhill PT, Doyle PS, On the coarse–graining of polymers into bead–spring chains, J. Non-Newton. Fluid Mech 122 (2004) 3–31.

[21] Underhill PT, Doyle PS, Development of bead–spring polymer models using the constant extension ensemble, J. Rheol 49 (2005) 963–987.

[22] Underhill PT, Doyle PS, Accuracy of bead–spring chains in strong flows, J. Non-Newton. Fluid Mech 145 (2007) 109–123.

[23] Öttinger HC, Stochastic Processes in Polymeric Fluids: Tools and Examples, Springer Science & Business Media, 2012.

[24] Herrchen M, Öttinger HC, A detailed comparison of various FENE dumbbell models, J. Non-Newton. Fluid Mech 68 (1) (1997) 17–42.

[25] Vincenzi D, Perlekar P, Biferale L, Toschi F, Impact of the Peterlin approximation on polymer dynamics in turbulent flows, Phys. Rev. E 92 (5) (2015) 053004. 10.1103/PhysRevE.92.053004 26651776

[26] Kuhn W, Grün F, Relationships between elastic constants and stretching double refraction of highly elastic substances, Kolloid-Z 101 (1942) 248–253.

[27] Warner HR Jr, Kinetic theory and rheology of dilute suspensions of finitely extendible dumbbells, Ind. Eng. Chem. Fundam 11 (3) (1972) 379–387.

[28] Treloar LG, The Physics of Rubber Elasticity, Oxford University Press, New York, 1975.

[29] Cohen A, A Padé approximant to the inverse Langevin function, Rheol. Acta 30 (1991) 270–273.

[30] Jedynak R, Approximation of the inverse Langevin function revisited, Rheol. Acta 54 (1) (2015) 29–39.

[31] Darabi E, Itskov M, A simple and accurate approximation of the inverse Langevin function, Rheol. Acta 54 (2015) 455–459.

[32] Kröger M, Simple and admissible and accurate approximants of the inverse Langevin and Brillouin functions, J. Non-Newton. Fluid Mech 223 (2015) 77–87.

[33] Jedynak R, A comprehensive study of the mathematical methods used to approximate the inverse Langevin function, Math. Mech. Solids 24 (7) (2019) 1992–2016.

[34] Howard RM, Analytical approximations for the inverse Langevin function via linearization, error approximation, and iteration, Rheol. Acta 59 (8) (2020) 521–544.

[35] Larson RG, Constitutive Equations for Polymer Melts and Solutions: Butterworths Series in Chemical Engineering, Butterworth-Heinemann, 2013.

[36] Li L, Larson RG, Sridhar T, Brownian dynamics simulations of dilute polystyrene solutions, J. Rheol 44 (2) (2000) 291–322.

[37] Itskov M, Dargazany R, Hörnes K, Taylor expansion of the inverse function with application to the Langevin function, Math. Mech. Solids 17 (7) (2012) 693–701.

[38] Nguessong AN, Beda T, Peyraut F, A new based error approach to approximate the inverse Langevin function, Rheol. Acta 53 (2014) 585–591.

[39] Rickaby S, Scott N, A comparison of limited-stretch models of rubber elasticity, Int. J. Non-Linear Mech 68 (2015) 71–86.

[40] Marchi BC, Arruda EM, An error-minimizing approach to inverse langevin approximations, Rheol. Acta 54 (2015) 887–902.

[41] Jedynak R, New facts concerning the approximation of the inverse langevin function, J. Non-Newton. Fluid Mech 249 (2017) 8–25.

[42] Sheikholeslami S, Aghdam M, A novel rational Padé approximation of the inverse Langevin function, in: 25th Annual International Conference on Mechanical Engineering, ISME2017, 2017, pp. 2–4.

[43] Benítez JM, Montáns FJ, A simple and efficient numerical procedure to compute the inverse Langevin function with high accuracy, J. Non-Newton. Fluid Mech 261 (2018) 153–163.

[44] Rickaby SR, Scott NH, On the complex singularities of the inverse Langevin function, IMA J. Appl. Math 83 (6) (2018) 1092–1116.

[45] Morovati V, Mohammadi H, Dargazany R, A generalized approach to generate optimized approximations of the inverse Langevin function, Math. Mech. Solids 24 (7) (2019) 2047–2059.

[46] Marchi BC, Arruda EM, Generalized error-minimizing, rational inverse Langevin approximations, Math. Mech. Solids 24 (6) (2019) 1630–1647.

[47] Barsan V, Inverses of Langevin, Brillouin and related functions: A status report, Rom. Rep. Phys 72 (2020) 109.

[48] Howard RM, Relationship between inverse Langevin function and *r*_0_–*r*_1_-Lambert W function, AppliedMath 4 (2) (2024) 743–762.

[49] Ammar A, Effect of the inverse langevin approximation on the solution of the Fokker–Planck equation of non-linear dilute polymer, J. Non-Newton. Fluid Mech 231 (2016) 1–5.

[50] Indei T, Koga T, Tanaka F, Theory of shear-thickening in transient networks of associating polymers, Macromol. Rapid Commun 26 (9) (2005) 701–706.

[51] Laso M, Öttinger HC, Calculation of viscoelastic flow using molecular models: the CONNFFESSIT approach, J. Non-Newton. Fluid Mech 47 (1993) 1–20.

[52] Öttinger HC, Van Den Brule B, Hulsen M, Brownian configuration fields and variance reduced CONNFFESSIT, J. Non-Newton. Fluid Mech 70 (3) (1997) 255–261.

[53] Prieto JL, Bermejo R, Laso M, A semi-Lagrangian micro–macro method for viscoelastic flow calculations, J. Non-Newton. Fluid Mech 165 (3–4) (2010) 120–135.

[54] Ewoldt RH, Hosoi AE, McKinley GH, New measures for characterizing nonlinear viscoelasticity in large amplitude oscillatory shear, J. Rheol 52 (6) (2008) 1427–1458.

[55] Hyun K, Wilhelm M, Klein CO, Cho K-S, Nam J-G, Ahn K-H, Lee S-J, Ewoldt RH, McKinley GH, A review of nonlinear oscillatory shear tests: Analysis and application of large amplitude oscillatory shear (LAOS), Prog. Polym. Sci 36 (12) (2011) 1697–1753.

[56] Anna SL, McKinley GH, Elasto-capillary thinning and breakup of model elastic liquids, J. Rheol 45 (2001) 115–138.

[57] Dinic J, Sharma V, Macromolecular relaxation, strain, and extensibility determine elastocapillary thinning and extensional viscosity of polymer solutions, Proc. Natl. Acad. Sci. USA 116 (2019) 8766–8774. 30979802 10.1073/pnas.1820277116 PMC6500132

[58] Prabhakar R, Gadkari S, Gopesh T, Shaw MJ, Influence of stretching induced self-concentration and self-dilution on coil-stretch hysteresis and capillary thinning of unentangled polymer solutions, J. Rheol 60 (2016) 345–366.

[59] Forest MG, Wang Q, Change-of-type behavior in viscoelastic slender jet models, Theor. Comput. Fluid Dyn 2 (1) (1990) 1–25.

[60] Eggers J, Nonlinear dynamics and breakup of free-surface flows, Rev. Modern Phys 69 (3) (1997) 865.

[61] Chase D, Cromer M, Roles of chain stretch and concentration gradients in capillary thinning of polymer solutions, Fluid Dyn. Res 56 (1) (2024) 015505.

[62] Guzel A, Layton W, Time filters increase accuracy of the fully implicit method, BIT Numer. Math 58 (2018) 301–315.

[63] Ardekani A, Sharma V, McKinley G, Dynamics of bead formation, filament thinning and breakup in weakly viscoelastic jets, J. Fluid Mech 665 (2010) 46–56.

