# Supplementary material for: Systematic comparison of inverse Langevin function approximations in stochastic dumbbell dynamics
Source: J Nonnewton Fluid Mech. Author manuscript; Available in PMC 2026 May 30. (PMC13215685; doi:10.1016/j.jnnfm.2026.105610)
Supplement: Supp Material [file NIHMS2175470-supplement-Supp_Material.pdf]

Supplementary Material

Computational Details and Numerical Schemes

This appendix provides details on the numerical methods used to solve the stochastic differential equations (SDEs) governing the dumbbell models, including the Brownian Configuration Field (BCF) formulation, time–integration scheme, model selection rationale, GPU implementation, and micro–macro coupling algorithm for non-viscometric flows.

Equations (4) and (16) form the basis for the stochastic integration of polymer dynamics under imposed flow for the FENE, Cohen, and RS models. To compute the polymeric stress, these stochastic differential equations (SDEs) are solved numerically for an ensemble of representative dumbbells. This is accomplished using the CONNFFESSIT framework (CONnection between Newtonian and Non–Newtonian Fluid Flow EStimated by Stochastic Simulation Technique) [1, 2], which couples microscopic bead–spring dynamics with macroscopic flow calculations. In this approach, many individual polymer configurations are evolved within a discretized flow domain, each experiencing local convection, deformation, and Brownian motion according to its governing SDE. Macroscopic quantities, such as the polymeric stress tensor, are then computed as ensemble averages over all simulated configurations.

While conceptually straightforward and capable of representing complex constitutive models, CONNFFESSIT simulations are computationally demanding. This high cost arises from the need to simulate a large ensemble of polymer chains to obtain statistically reliable averages—particularly in complex flow geometries or at high Weissenberg numbers. To reduce both computational expense and statistical noise, various variance–reduction strategies have been proposed. Early approaches include the control variate method introduced by Öttinger et al. [3], which employs an equilibrium stochastic variable as a control in a parallel process simulation, and the Brownian Configuration Field (BCF) method developed by Hulsen et al. [4]. In the present study, we adopt the BCF method as a convenient field-based route for the coupled simulations considered here, and describe it in detail in the following section.

Brownian Configuration Field Formulation

Rather than tracking individual polymer molecules, the Brownian Configuration Field (BCF) method provides a continuum description based on an ensemble of spatially continuous configuration fields. These fields represent the polymers' internal degrees of freedom and are convected, deformed, and diffused by the flow in a manner consistent with the underlying stochastic dynamics. As demonstrated by Hulsen et al. [4] for an Oldroyd–B fluid flowing past a cylinder, the BCF approach achieves excellent agreement with macroscopic simulations while exhibiting greater numerical stability, particularly at high Deborah numbers. It also produces smooth stress fields and controls statistical error through a single parameter that specifies the number of configuration fields employed.

Within the BCF framework, the evolution of a configuration field is governed by

$$d\mathbf{Q}(\mathbf{x},t) = \left[-\mathbf{u}(\mathbf{x},t)\cdot\nabla\mathbf{Q}(\mathbf{x},t) + \kappa(\mathbf{x},t)\cdot\mathbf{Q}(\mathbf{x},t) - \frac{F(\mathbf{Q}(\mathbf{x},t))}{2De}\right]dt + \frac{d\mathbf{W}(t)}{\sqrt{De}}, \tag{S1}$$

where $\kappa(\mathbf{x},t) = (\nabla\mathbf{u})^{\intercal}$.

Semi–implicit time–integration scheme

The evolution of the configuration field is governed by Eqn. (S1), where the nonlinearity arises from the spring force term $\mathbf{F}(\mathbf{Q})$. For the FENE, Cohen, and RS approximations, this force takes the unified form,

$$\mathbf{F}(\mathbf{Q}) = \mathbf{Q}\left(1 + \frac{2A}{3}\frac{Q^2/b}{1-Q^2/b}\right), \tag{S2}$$

where $Q = \|\mathbf{Q}\|$, A is the model–specific parameter ($A = 3/2$ for FENE, $A = 1$ for Cohen, and $A = 9/10$ for RS), and the extensibility parameter is defined as $b = HQ^2_{\max}/(k_BT)$, with H the Hookean spring constant, $Q_{\max}$ the maximum chain extension, k_B Boltzmann's constant, and T the temperature.

Due to this nonlinearity in $\mathbf{F}(\mathbf{Q})$, a semi–implicit, first–order algorithm is required rather than a forward Euler scheme [1]. The update employs a two–step operator–splitting procedure that treats explicit linear terms separately from the implicit nonlinear elastic term for stability.

Step I: Explicit Integration (Intermediate Vector $\overline{\mathbf{Q}}$)

The first step integrates convection, linear deformation, and Brownian motion explicitly to compute an intermediate vector $\overline{\mathbf{Q}}(\mathbf{x}, t_{j+1})$:

$$\begin{aligned}\overline{\mathbf{Q}}(\mathbf{x}, t_{j+1}) &= \mathbf{Q}(\mathbf{x}, t_j) + \left[-\mathbf{u}(\mathbf{x}, t_j) \cdot \nabla \mathbf{Q}(\mathbf{x}, t_j) + \kappa(\mathbf{x}, t_j) \cdot \mathbf{Q}(\mathbf{x}, t_j) - \alpha_j \mathbf{Q}(\mathbf{x}, t_j) \right] \Delta t_j \\ &\quad + \frac{1}{\sqrt{\mathrm{De}}} \Delta \mathbf{W}_j, \end{aligned} \tag{S3}$$

where α_j is the time–dependent, non–linear coefficient associated with the spring force at the time step t_j, given by

$$\alpha_j = \frac{1}{2\mathrm{De}} \left(1 + \frac{2A}{3} \frac{Q(\mathbf{x}, t_j)^2 / b}{1 - Q(\mathbf{x}, t_j)^2 / b} \right). \tag{S4}$$

Step II: Semi–implicit Non–linear Elasticity (Final Vector $\mathbf{Q}_{j+1}$)

The second step introduces the nonlinear spring force semi–implicitly. Define:

$$\begin{aligned}\mathbf{RHS}_{j+1} &= \mathbf{Q}(\mathbf{x}, t_j) + \frac{1}{\sqrt{\mathrm{De}}} \Delta \mathbf{W}_j + \frac{\Delta t_j}{2} \Big[-\mathbf{u}(\mathbf{x}, t_{j+1}) \cdot \nabla \overline{\mathbf{Q}}(\mathbf{x}, t_{j+1}) \\ &\quad -\mathbf{u}(\mathbf{x}, t_j) \cdot \nabla \mathbf{Q}(\mathbf{x}, t_j) + \kappa(\mathbf{x}, t_{j+1}) \cdot \overline{\mathbf{Q}}(\mathbf{x}, t_{j+1}) + \kappa(\mathbf{x}, t_j) \cdot \mathbf{Q}(\mathbf{x}, t_j) - \alpha_j \mathbf{Q}(\mathbf{x}, t_j) \Big]. \end{aligned}$$

The implicit update is then given by:

$$\left[1 + \frac{\alpha_j \Delta t_j}{2} \right] \mathbf{Q}(\mathbf{x}, t_{j+1}) = \mathbf{RHS}_{j+1}. \tag{S5}$$

This system is solved in two parts: magnitude and direction.

Magnitude Update Taking the norm on Eqn. (S5) reduces the problem to solving a scalar cubic equation for the magnitude $Q_{j+1} = \|\mathbf{Q}(\mathbf{x}, t_{j+1})\|$:

$$Q_{j+1}^3 - P Q_{j+1}^2 - b \left[1 + \frac{\Delta t_j}{4\mathrm{De}} \left(1 - \frac{2A}{3} \right) \right] Q_{j+1} + bP = 0, \tag{S6}$$

where $P = \|\mathbf{RHS}_{j+1}\|$ is the magnitude of the right–hand side vector. It has been shown that, in the case of FENE ($A = 3/2$), this cubic equation has one real root in the physical interval $\left(0, \sqrt{b}\right)$ [1, 5]; we extended this finding to $A = 1, 9/10$ in .

Direction Update The implicit operator affects only the magnitude, so the final vector retains the direction of $\mathbf{RHS}_{j+1}$:

$$\mathbf{Q}(\mathbf{x}, t_{j+1}) = \frac{Q_{j+1}}{P} \mathbf{RHS}_{j+1}. \tag{S7}$$

Finally, the scheme requires flow fields, $\mathbf{u}$ and $\kappa = (\nabla \mathbf{u})^\intercal$, at time t_{j+1}, which are unknown for non–viscometric flows. In our computations, $\mathbf{u}(t_{j+1})$ is found by a second–order extrapolation following [5]:

$$\mathbf{u}(\mathbf{x}, t_{j+1}) = 2\mathbf{u}(\mathbf{x}, t_j) - \mathbf{u}(\mathbf{x}, t_{j-1}). \tag{S8}$$

Justification of model selection

Although many approximants to the inverse Langevin function exist [6, 7, 8, 9, 10, 11, 12, 13], we intentionally restrict our study to the three models introduced above (FENE, Cohen, and Rickaby–Scott). There are three complementary reasons for this choice.

First, the FENE form is by far the most widely used finite–extensibility law in polymer physics and serves as the natural baseline for comparisons. Second, the Cohen and Rickaby–Scott approximants are Padé–type refinements of the inverse Langevin function that (i) substantially reduce approximation error relative to FENE while (ii) preserving the same rational structure. In particular, all three approximants can be written in the unified form of Eqn. (16), which makes them physically and algebraically comparable.

Third — and most important for the numerical study presented here — the unified form in Eqn. (16) enables a common predictor–corrector semi–implicit time integration, reducing the implicit corrector step to the scalar cubic in Eqn. (S6). This ensures that differences in stochastic dynamics and macroscopic stresses arise from the approximants themselves rather than from variations in numerical treatment. Other ILF approximations do not share this property and would require different solver strategies, complicating comparisons.

GPU implementation

The stochastic nature of the dumbbell and Brownian Configuration Field (BCF) formulations makes them ideally suited to parallel computation. In this work, mesoscale calculations are performed on graphics processing units (GPUs), which allow millions of independent SDE realizations to be solved concurrently. Because the dynamics of individual dumbbells are non–interacting, this problem is trivially parallelizable. However, incorporating hydrodynamic interactions would couple the dynamics of individual chains and greatly increase computational cost, so our study is limited to models that remain trivially parallelizable on GPU architectures.

Micro–Macro Coupling Algorithm for Non–Viscometric Flows

Figure S1 summarizes the algorithm used to couple the micro– and macroscale equations. The microscopic stochastic differential equations are integrated using the semi–implicit time–stepping scheme described in Section , without modification for the capillary–thinning simulations.

At each macroscopic time step, the following sequence is performed:

1. Evaluate the macroscopic velocity field $\mathbf{u}$ and velocity–gradient tensor $\nabla\mathbf{u}$ at all grid points.
2. Use these quantities to compute the convective term $\mathbf{u}\cdot\nabla\mathbf{Q}$ for each configuration field.
3. Advance the microscopic stochastic equations over one macroscopic time step using multiple micro time steps ($\Delta t_{\text{micro}} \ll \Delta t_{\text{macro}}$), while holding the macroscopic fields fixed.
4. Enforce boundary conditions on the configuration fields $\mathbf{Q}$.
5. Compute the polymeric stress tensor from the updated microstructure.
6. If mesh adaptation is required, update the computational mesh and interpolate the polymeric stress onto the new grid.
7. Solve the macroscopic equations for the filament radius and velocity using the updated polymeric stress.
8. If mesh refinement occurred, interpolate the macroscopic velocity field onto the uniform reference grid.

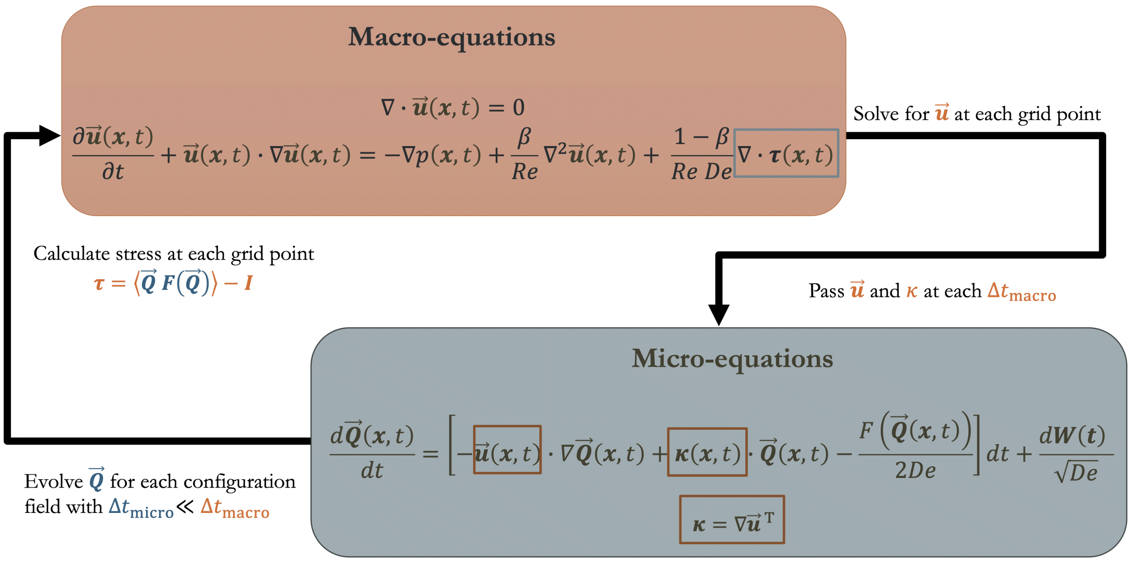

Figure S1: Schematic of the micro–macro coupling algorithm, illustrating the exchange of velocity and velocity–gradient information from the macroscopic solver to the microscopic solver, and the feedback of polymeric stress to the macroscopic equations.

Existence and uniqueness of the cubic root in $(0, \sqrt{b})$

In Section the semi–implicit update for the configuration magnitude $Q_{j+1} = \|\mathbf{Q}(\mathbf{x}, t_{j+1})\|$ leads to

$$x^3 - Px^2 - b\left[1 + \frac{\Delta t}{4De}\left(1 - \frac{2A}{3}\right)\right]x + bP = 0, \tag{S9}$$

with physical parameters $P, b, \Delta t, De > 0$ and model constant $A = \frac{3}{2}$ (FENE), $A = 1$ (Cohen), or $A = \frac{9}{10}$ (Rickaby–Scott). Here $P = \|\mathrm{RHS}(\mathbf{x})\|$ is the predictor magnitude and $\sqrt{b}$ is the nondimensional maximum chain extension.

Define $C = 1 + \frac{\Delta t}{4De}\left(1 - \frac{2A}{3}\right)$.

Model	A	C
FENE	$\frac{3}{2}$	1
Cohen	1	$1 + \dfrac{\Delta t}{12De}$
RS	$\frac{9}{10}$	$1 + \dfrac{\Delta t}{10De}$

Since all parameters are positive and $A \leq \frac{3}{2}$ for the models considered, we have $C \geq 1$.

The goal is to show that the scalar equation

$$f(x) = x^3 - Px^2 - bCx + bP = 0$$

has exactly one real root in $(0, \sqrt{b})$ under these physically admissible conditions.

Existence. At the interval endpoints,

$$f(0) = bP > 0, \qquad f(\sqrt{b}) = b\sqrt{b}(1 - C) \leq 0,$$

since $C \geq 1$. Because $f(x)$ is continuous on $(0, \sqrt{b})$, the Intermediate Value Theorem guarantees at least one root in $(0, \sqrt{b})$.

Uniqueness. The derivative and second derivative of f are

$$f'(x) = 3x^2 - 2Px - bC, \qquad f''(x) = 6x - 2P.$$

Since $f''(x)$ is linear and $f'''(x) = 6 > 0$, the curvature of f increases monotonically with x. Thus, $f'(x)$ is a convex quadratic that can have at most one positive root. Solving $f'(x) = 0$ gives

$$x_{\min} = \frac{P + \sqrt{P^2 + 3bC}}{3} > 0,$$

which corresponds to the unique local minimum of $f(x)$, since $f''(x_{\min}) = 6x_{\min} - 2P > 0$. Therefore,

$$f(x) \text{ is strictly decreasing for } x < x_{\min}, \quad \text{and strictly increasing for } x > x_{\min}.$$

We also have $f(0) = bP > 0$ and $f'(0) = -bC < 0$, so $f(x)$ initially decreases from a positive value. Because $\lim_{x\to\infty} f'(x) = +\infty$, $f'(x)$ changes sign exactly once, and $f(x)$ increases monotonically thereafter. Hence, $f(x)$ can have at most one turning point for $x > 0$—a single local minimum at $x_{\min}$.

Asymptotic eigenvalue behavior near full extension

To quantify the stiffness of the governing equations as chains approach full extension ($Q^2 \to b$) and compare the three ILF approximations, we linearize the deterministic part of Eqn. (4a) around a state $\mathbf{Q}$ (ignoring the stochastic term):

$$\mu(\mathbf{Q}) = (\nabla \mathbf{u})^{\mathsf{T}} \cdot \mathbf{Q} - \frac{1}{2De} f(Q)\mathbf{Q}, \quad f(Q) = 1 + \frac{2A}{3} \cdot \frac{Q^2/b}{1 - Q^2/b}, \quad Q^2 = Q_x^2 + Q_y^2 \tag{S10}$$

We restrict our analysis to the two–dimensional case in the (x, y)–plane, where $Q^2 = Q_x^2 + Q_y^2$, as this captures the essential stiffness behavior while simplifying the algebra.

For uniaxial extension, the velocity gradient is:

$$(\nabla \boldsymbol{u})^{\mathsf{T}} = \begin{pmatrix} \dot{\epsilon}_0 & 0 \\ 0 & -\frac{\dot{\epsilon}_0}{2} \end{pmatrix} \tag{S11}$$

So that the Jacobian of the flow term is:

$$\mathbf{J}_{\text{flow}} = \begin{pmatrix} \dot{\epsilon}_0 & 0 \\ 0 & -\frac{\dot{\epsilon}_0}{2} \end{pmatrix}. \tag{S12}$$

For the force term we have,

$$\frac{\partial f}{\partial Q_x} = \frac{4AQ_x b}{3(b - Q^2)^2}, \quad \frac{\partial f}{\partial Q_y} = \frac{4AQ_y b}{3(b - Q^2)^2}, \tag{S13}$$

So that, the Jacobian of the force term is then

$$\begin{aligned} \mathbf{J}_{\text{force}} &= -\frac{1}{2De}\left(f(Q)I + \boldsymbol{Q} \otimes \nabla f\right) \\ &= -\frac{1}{2De}\left(f(Q)\begin{pmatrix} 1 & 0 \\ 0 & 1 \end{pmatrix} + \frac{4Ab}{3(b - Q^2)^2}\begin{pmatrix} Q_x^2 & Q_x Q_y \\ Q_y Q_x & Q_y^2 \end{pmatrix}\right) \end{aligned} \tag{S14}$$

The full Jacobian is then given by

$$\mathbf{J} = \mathbf{J}_{\text{flow}} + \mathbf{J}_{\text{force}} = \begin{pmatrix} \dot{\epsilon}_0 - \frac{f(Q)}{2De} & 0 \\ 0 & -\frac{\dot{\epsilon}_0}{2} - \frac{f(Q)}{2De} \end{pmatrix} - \frac{1}{2De}\frac{4Ab}{3(b - Q^2)^2}\begin{pmatrix} Q_x^2 & Q_x Q_y \\ Q_y Q_x & Q_y^2 \end{pmatrix} \tag{S15}$$

The characteristic polynomial is

$$\lambda^2 - \mathrm{Tr}(J)\lambda + \det(J) = 0 \tag{S16}$$

Where:

$$\begin{aligned} \mathrm{Tr}(J) &= \dot{\epsilon}_0 - \frac{\dot{\epsilon}_0}{2} - \frac{f(Q)}{De} - \frac{1}{De}\frac{2Ab}{3(b - Q^2)^2} Q^2 \\ &= \frac{\dot{\epsilon}_0}{2} - \frac{1}{De}\left(1 - \frac{2AQ^4}{3(b - Q^2)^2}\right) \\ \det(J) &= \left(\dot{\epsilon}_0 - \frac{f(Q)}{2De}\right)\left(-\frac{\dot{\epsilon}_0}{2} - \frac{f(Q)}{2De}\right) \\ &\quad - \frac{2Ab}{3De(b - Q^2)^2}\left[\left(\dot{\epsilon}_0 - \frac{f(Q)}{2De}\right) Q_y^2 + \left(-\frac{\dot{\epsilon}_0}{2} - \frac{f(Q)}{2De}\right) Q_x^2\right]. \end{aligned}$$

As $Q^2 \to b$, the force term dominates due to the $(b - Q^2)^{-2}$ singularity, while the flow term $\dot{\epsilon}_0$ remains $O(1)$. So that,

$$\begin{aligned} \mathrm{Tr}(\mathbf{J}) &\approx \frac{2Ab^2}{3De(b - Q^2)^2} \\ \det(\mathbf{J}) &\approx \frac{2A^2 b^3}{9De^2(b - Q^2)^3}. \end{aligned}$$

The leading–order approximation of the characteristic polynomial is therefore

$$\lambda^2 - \frac{2Ab^2}{3De(b - Q^2)^2}\lambda + \frac{2A^2 b^3}{9De^2(b - Q^2)^3} \approx 0,$$

and the roots are

$$\lambda_+ \approx \frac{2Ab^2}{3De(b-Q^2)^2}, \quad \lambda_- \approx \frac{Ab}{3De(b-Q^2)}. \tag{S17}$$

For each of the three models, this yields

$$\begin{aligned} A=1: &\quad \lambda_+ \approx \frac{2b^2}{3De(b-Q^2)^2}, \quad \lambda_- \approx \frac{b}{3De(b-Q^2)}, \\ A=\frac{3}{2}: &\quad \lambda_+ \approx \frac{b^2}{De(b-Q^2)^2}, \quad \lambda_- \approx \frac{b}{2De(b-Q^2)}, \\ A=\frac{9}{10}: &\quad \lambda_+ \approx \frac{3b^2}{5De(b-Q^2)^2}, \quad \lambda_- \approx \frac{3b}{10De(b-Q^2)}. \end{aligned} \tag{S18}$$

The dominant eigenvalue λ_+ controls the stiffness of the system. Table S1 summarizes the numerical stiffness coefficients for each model.

Table S1: Numerical stiffness coefficients from the pre–factor of $(b-Q^2)^{-2}$ in λ_+ for the three ILF approximations.

Model	A	Stiffness Coefficient
FENE	$3/2$	1
Cohen	1	$2/3 \approx 0.67$
RS	$9/10$	$3/5 = 0.60$

Cohen and RS exhibit comparable stiffness coefficients and should have similar convergence characteristics. In contrast, FENE's stiffness coefficient is 1.5–1.67 times larger, indicating that FENE is approximately 50–67% numerically stiffer than Cohen and RS. Consequently, FENE requires time steps that are approximately 33–40% smaller near full extension to maintain numerical stability.

Distribution of Dumbbell Stretching in LAOS

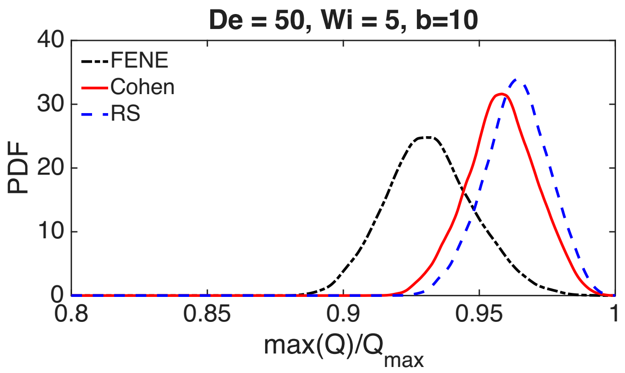

(a)

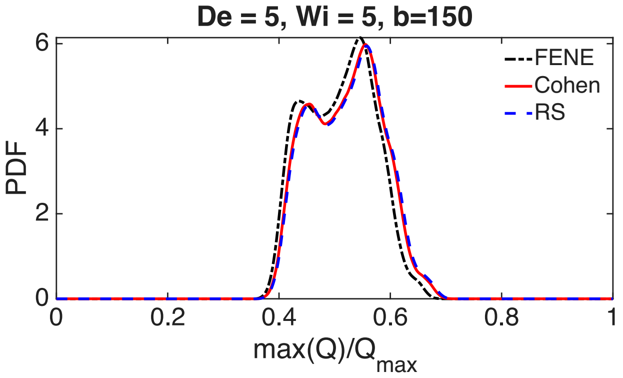

(b)

Figure S2: Probability Density Function (PDF) of the maximum dumbbell extension normalized by the finite extensibility limit ($\max(Q)/Q_{\max}$) for the entire duration of the LAOS simulation. The distributions account for the maximum configuration values (Q_{r_max}) recorded at every macro time step throughout the evolution of the system. Results are compared across three spring force models: FENE (dash-dotted black line), Cohen (solid red line), and RS (dashed blue line). Simulation parameters: (a) $De = 50, Wi = 5, b = 10$; (b) $De = 5, Wi = 5, b = 150$. All PDFs were constructed using a kernel density estimation with a triangular kernel.

References

[1] H. C. Öttinger, Stochastic processes in polymeric fluids: tools and examples, Springer Science & Business Media, 2012.

[2] M. Laso, H. C. Öttinger, Calculation of viscoelastic flow using molecular models: the CONNFFESSIT approach, Journal of Non-Newtonian Fluid Mechanics 47 (1993) 1–20.

[3] H. C. Öttinger, B. Van Den Brule, M. Hulsen, Brownian configuration fields and variance reduced CONNFFESSIT, Journal of Non-Newtonian Fluid Mechanics 70 (3) (1997) 255–261.

[4] M. Hulsen, A. Van Heel, B. Van Den Brule, Simulation of viscoelastic flows using Brownian configuration fields, Journal of Non-Newtonian Fluid Mechanics 70 (1-2) (1997) 79–101.

[5] J. L. Prieto, R. Bermejo, M. Laso, A semi-Lagrangian micro–macro method for viscoelastic flow calculations, Journal of Non-Newtonian Fluid Mechanics 165 (3-4) (2010) 120–135.

[6] M. Kröger, Simple models for complex nonequilibrium fluids, Physics Reports 390 (6) (2004) 453–551.

[7] A. Cohen, A Padé approximant to the inverse Langevin function, Rheologica Acta 30 (1991) 270–273.

[8] E. Darabi, M. Itskov, A simple and accurate approximation of the inverse Langevin function, Rheologica Acta 54 (2015) 455–459.

[9] M. Kröger, Simple, admissible, and accurate approximants of the inverse Langevin and Brillouin functions, Journal of Non-Newtonian Fluid Mechanics 223 (2015) 77–87.

[10] R. M. Howard, Analytical approximations for the inverse Langevin function via linearization, error approximation, and iteration, Rheologica Acta 59 (8) (2020) 521–544.

[11] M. Itskov, R. Dargazany, K. Hörnes, Taylor expansion of the inverse function with application to the Langevin function, Mathematics and Mechanics of Solids 17 (7) (2012) 693–701.

[12] S. Rickaby, N. Scott, A comparison of limited-stretch models of rubber elasticity, International Journal of Non-Linear Mechanics 68 (2015) 71–86.

[13] J. M. Benítez, F. J. Montáns, A simple and efficient numerical procedure to compute the inverse Langevin function with high accuracy, Journal of Non-Newtonian Fluid Mechanics 261 (2018) 153–163.
